# Oxygen evolution catalysts by self-assembly of MS_2_ (M = Mo, W) and Ni_3_Fe-LDH nanosheets

**DOI:** 10.1039/d6ra07498g

**Published:** 2026-10-07

**Authors:** Matthew R. L. Williams, Yang Li, Roland C. Turnell-Ritson, Dermot O'Hare

**Affiliations:** a Chemistry Research Laboratory, Department of Chemistry, University of Oxford Mansfield Road Oxford OX1 3TA UK dermot.ohare@chem.ox.ac.uk +44(0)1865 272686

## Abstract

Layered double hydroxides (LDHs) have been demonstrated as high-activity electrocatalysts for the oxygen evolution reaction (OER) but their performance is limited by their inherently low electrical conductivity. This can be mitigated by the formation of a hybrid material composed of transition metal dichalcogenide (TMD) layers interleaved between LDH sheets. Here, we report an improved synthesis of TMD/LDH hybrid materials using a post-synthetic aqueous miscible organic solvent treatment (AMOST) on Ni_3_Fe-borate-LDH to yield predominantly mono/few-layer LDH nanosheets. These LDH nanosheets when combined with either MoS_2_ or WS_2_ monolayers create MoS_2_/Ni_3_Fe-LDH and WS_2_/Ni_3_Fe-LDH hybrid materials, respectively. The hybrid materials prepared in this way exhibit improved electrocatalytic performance towards the OER, with low overpotentials at 10 mA cm^−2^ of 258 and 248 mV, respectively. Over 12 h chronopotentiometric testing, both hybrids show lower potential drift than pristine Ni_3_Fe-borate-LDH, while separate extended measurements maintained operation at 10 mA cm^−2^ for approximately 20 h.

## Abstract 2

Layered double hydroxides (LDHs) have been demonstrated as high-activity electrocatalysts for the oxygen evolution reaction (OER), but their performance can be limited by their relatively low electrical conductivity.

## Graphical abstract

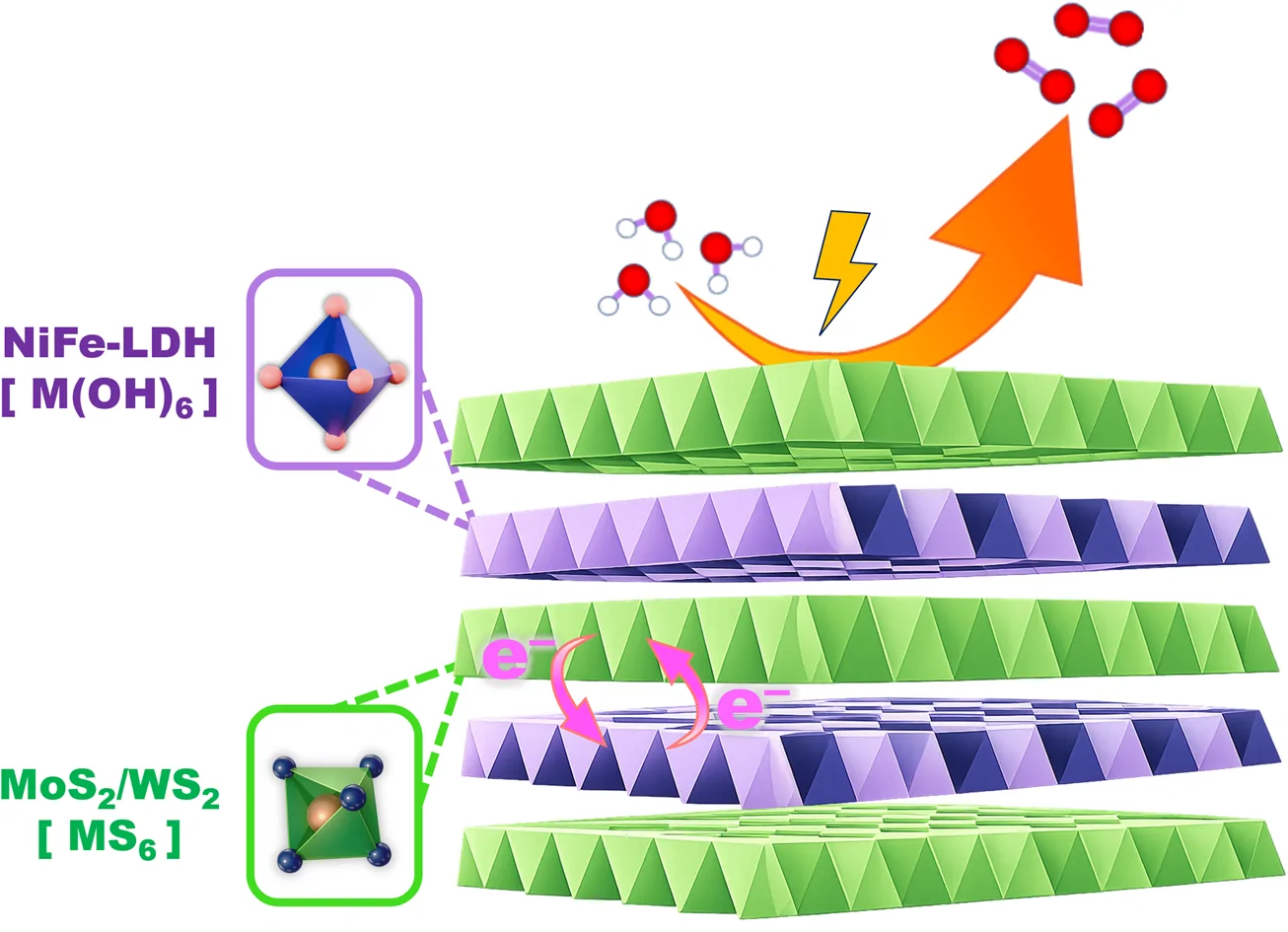

## Introduction

Energy production and transport are the dominant sources of anthropogenic greenhouse gas emissions, with CO_2_ being a major contributor to climate change.^1^ To reduce CO_2_ emissions and possibly mitigate the long-term implications of climate change, 30 TW of carbon-free power is required over the next two decades.^2^ The development of sustainable fuels which can be prepared from renewable energy is therefore a current and vital problem.

Hydrogen is a promising alternative energy vector with a high gravimetric energy density and, importantly, emits only water upon combustion.^3^ In order to produce hydrogen, electrolysis of water presents an attractive option (Fig. 1). The process is comprised of two half-cell reactions: the hydrogen evolution reaction (HER) occurs at the cathode, and the oxygen evolution reaction (OER) takes place at the anode. Under alkaline conditions, they can be represented as:HER: 2H_2_O + 2e^−^ → H_2_ + 2OH^−^OER: 4OH^−^ → O_2_ + 2H_2_O + 4e^−^

**Fig. 1:**
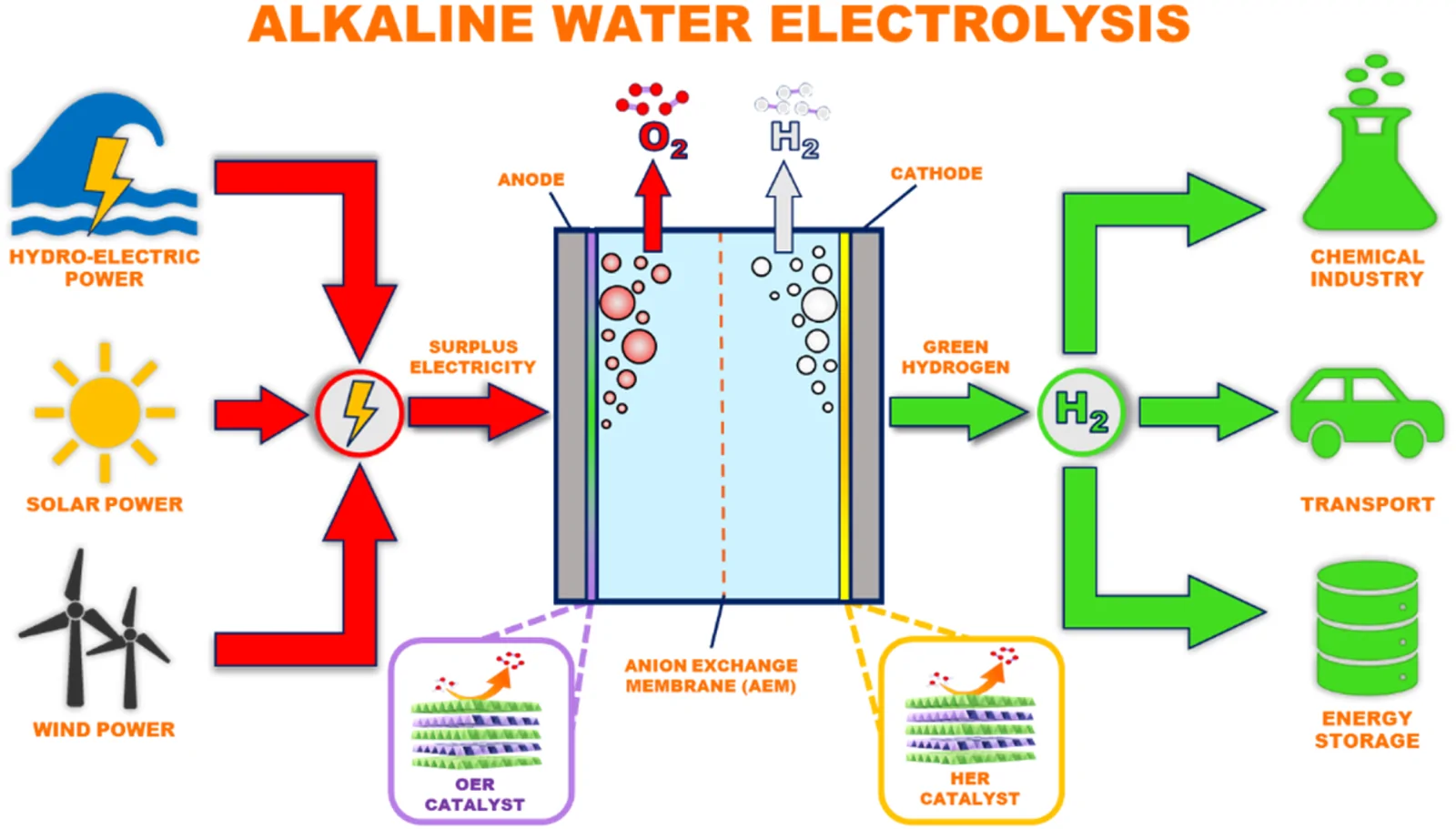
Schematic diagram to represent the alkaline water electrolysis system, from renewable energy inputs, to generate ‘green’ hydrogen for use in transportation, energy storage, and the chemical industry.

The OER, with its complex four-electron–proton-coupled reaction pathway, suffers from sluggish kinetics that necessitate high overpotentials to drive the reaction at appreciable current densities. This energy requirement limits the overall efficiency of water splitting. Appropriate electrocatalysts lower this energy barrier, traditionally relying on the use of scarce, expensive noble metal oxides, such as RuO_2_ and IrO_2_.^4^ Recently, the use of more Earth-abundant transition metals (*e.g.*, Mn, Fe, Co, and Ni) has offered a promising, scalable, and low-cost alternative without compromising on electrochemical activity.^5^

Layered double hydroxides (LDHs) are a class of synthetic clay which may be most frequently represented by the formula: [M_1−*x*_^2+^M_*x*_^3+^(OH)_2_]^*x*+^(A_*x*/*n*_^*n*−^)·*m*H_2_O.^6^ They are lamellar materials structurally derived from the brucite (Mg(OH)_2_) structure, wherein a fraction, *x*, of the divalent cations have been substituted for M^3+^ cations. For a stable LDH phase, *x* normally lies in the range 0.18–0.33.^7^ The resultant hydroxide layers possess a positive charge, necessitating the intercalation of charge-balancing interlayer anions, A^*n*−^. Polar solvent molecules can further stabilise the lamellar structure through an extensive hydrogen bonding network. LDHs are facile to prepare *via* co-precipitation of appropriate metal salts into a basic solution, and have high compositional tuneability, with a variety of applications spanning thermocatalysis,^8,9^ photocatalysis,^10,11^ and electrocatalysis.^12–14^ LDHs comprised of Earth-abundant transition metals have shown high electrocatalytic activity towards OER, with their performance strongly influenced by composition, nanosheet morphology, and the electronic environment of the metal centres.^15–17^ For example, Zhang *et al.* demonstrated that trimetallic NiCoFe-LDH nanosheets exhibit enhanced OER activity and charge-transfer characteristics relative to related bimetallic NiFe- and NiCo-LDH systems.^18^ Ni_*x*_Fe-LDHs are some of the most active materials for alkaline water electrolysis, with extensive studies into their electrocatalytic OER performance.^4,16,19–22^ Although they possess high intrinsic activity, their performance is limited by restricted access to active sites and intrinsically low electrical conductivity of LDHs.^23^ Improvements to Ni_*x*_Fe-LDH electrocatalysts must confront these challenges to yield materials with lower overpotentials and improved charge-transfer kinetics.

One strategy to address the issue of charge-transfer kinetics is to construct hybrid materials combining LDHs with more conductive substrates. Liu *et al.* and Dai *et al.* have grown LDHs on conductive substrates, such as carbon nanotubes or nickel foam, which have led to lower OER overpotentials compared to the pristine LDHs.^16,24^ Ma *et al.* have used 2D conductive materials, such as graphene, interstratified between the LDH layers to improve the overall conductivity of the material, to a similar effect.^25^ Transition metal dichalcogenides (TMDs), such as MoS_2_, have also been used to create hybrid materials through the electrostatic self-assembly of oppositely charged TMD and LDH nanosheets.^26,27^ These TMD/LDH hybrid materials have demonstrated excellent activity towards both the OER and the HER, presenting an attractive bifunctional catalyst for water splitting. However, preparation methods require laborious LDH anion-exchange and delamination *via* dispersion in formamide to achieve LDH monolayers.^26,27^ There has also been limited exploration of other TMDs, such as WS_2_ or MoSe_2_. Compositional variation of the TMD could lead to tuning of the LDH band structure, increasing the intrinsic activity of the hybrid materials.^28,29^

In our previous work, we conclusively demonstrated that Mg_3_Al-borate-LDHs can be exfoliated to deliver individual nanosheets using the aqueous miscible organic solvent treatment (AMOST) approach. This methodology yields dispersible powders containing LDH monolayers.^30,31^ Herein, we report the use of AMOST on Ni_3_Fe-borate-LDH to achieve predominantly monolayer Ni_3_Fe-LDH. The charged LDH mono/few-layer nanosheets can undergo solution-phase electrostatic self-assembly with TMD nanosheets (MoS_2_ or WS_2_) to form TMD/LDH hybrid materials: MoS_2_/Ni_3_Fe-LDH and WS_2_/Ni_3_Fe-LDH (Fig. 2). Structural studies, surface properties, and electrocatalytic OER activity of the hybrid materials were investigated. Both hybrids exhibit excellent OER activity, with significantly lower overpotentials required to achieve a current density of 10 mA cm^−2^ (*η*_10_) relative to pristine Ni_3_Fe-LDH.

**Fig. 2:**
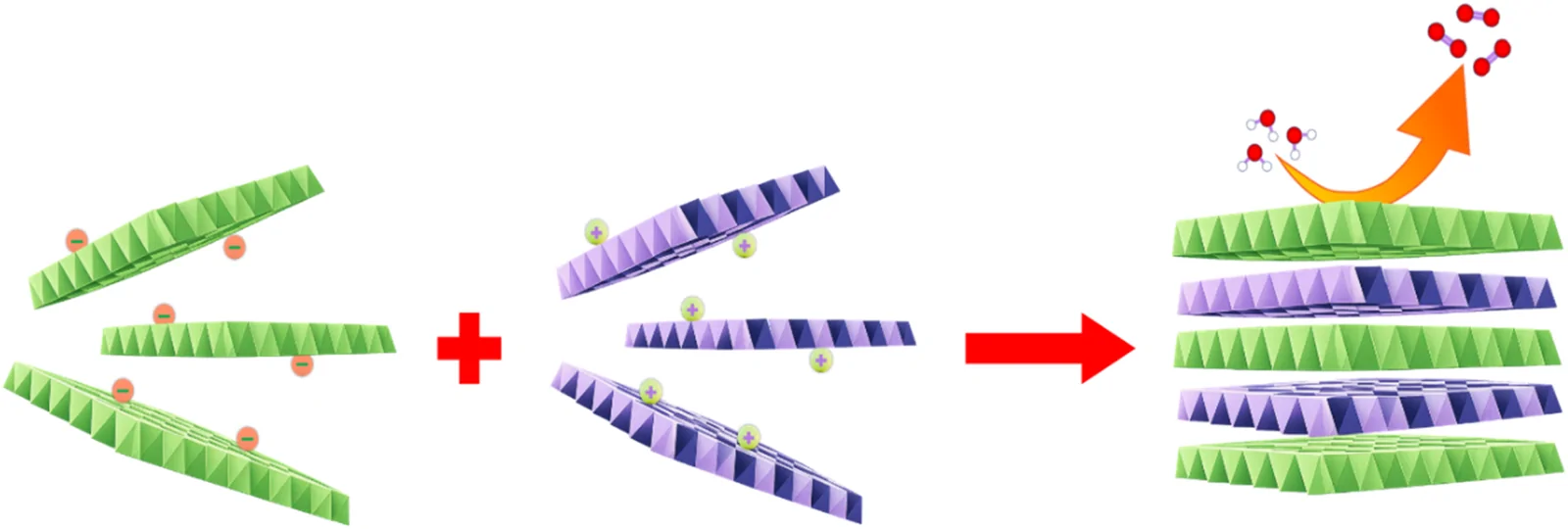
Schematic visualisation of the synthesis of the TMD/LDH hybrid by the electrostatic self-assembly of oppositely charged monolayer nanosheets of the TMD (green, negative charge) and the LDH (purple, positive charge) for OER electrocatalysis.

## Experimental

### Synthesis of C-Ni_3_Fe-borate LDH and AMO-Ni_3_Fe-borate LDH

Into a solution of B(OH)_3_ (5.87 g, 0.094 mol) in deionised-(dI-)decarbonated-(dC-)water (50 mL) were added dropwise at 115 mL h^−1^ a mixture of Ni(NO_3_)_2_·6H_2_O (10.90 g, 0.0375 mol) and Fe(NO_3_)_3_·9H_2_O (5.05 g, 0.0125 mol) in 50 mL of dI-dC-water under a N_2_ atmosphere with stirring at 800 rpm. A constant pH = 9 was maintained manually using dropwise addition of a solution of NaOH (1 M). The sample was then aged for 24 h at 65 °C, with stirring at 600 rpm, separated *via* vacuum filtration, and washed using dI-dC-water until pH ∼7 and dried at 65 °C to yield a conventional LDH (C-Ni_3_Fe-borate LDH). A portion of the wet cake sample was subjected to AMOST; first by washing with isopropyl alcohol (IPA) (250 mL) and subsequently dispersing in IPA (400 mL) with stirring at 1400 rpm for 2 h, before filtration and drying in a vacuum oven overnight at 65 °C to give AMO-Ni_3_Fe-borate LDH.

### Synthesis of exfoliated MoS_2_ – “Li_*x*_MoS_2_ nanosheets”

Bulk MoS_2_ (1.00 g, 6.25 mmol) was added to an ampoule and evacuated for 1 h. 40 mL of 2.5 M *n*BuLi (in hexane) was added under N_2_ and the mixture stirred at 800 rpm for 72 h. After 72 h, the mixture was washed and filtered under N_2_ with 3 × 50 mL dry hexane. The formed Li_*x*_MoS_2_ was dispersed in 200 mL dI-dC-water and sonicated for 1 h. The sample was then centrifuged at 2000 rpm for 10 min, and the resulting supernatant was collected by centrifugation at 9000 rpm for 15 min. The solid was then re-dispersed in dI-dC-water.

### Synthesis of exfoliated WS_2_ – “Li_*x*_WS_2_ nanosheets”

Bulk WS_2_ (1.55 g, 6.25 mmol) was added to an ampoule and evacuated for 1 h. 40 mL of 2.5 M *n*BuLi (in hexane) was added under N_2_ and the mixture stirred at 800 rpm for 96 h at 50 °C. After 96 h, the mixture was washed and filtered under N_2_ with 3 × 50 mL dry hexane. The as-formed Li_*x*_WS_2_ was dispersed in 200 mL dI-dC-water and sonicated for 1 h. The sample was then centrifuged at 2000 rpm for 10 min, and the resulting supernatant was collected by centrifugation at 9000 rpm for 15 min. The solid was then re-dispersed in dI-dC-water.

### Synthesis of MoS_2_/Ni_3_Fe-LDH and WS_2_/Ni_3_Fe-LDH hybrid materials

For the MoS_2_/Ni_3_Fe-LDH – Dry AMO-Ni_3_Fe-borate-LDH powder (0.3 g) was dispersed in 20 mL of formamide. 10 mL of this mixture was then added dropwise to 100 mL of MoS_2_ in dI-dC-water (0.27 mg mL^−1^) at a rate of 20 mL h^−1^ under a N_2_ atmosphere at 300 rpm. After 2 h, the mixture was centrifuged at 6000 rpm for 10 min, washed with formamide, and then ethanol, before drying in a vacuum oven at 65 °C.

For WS_2_/Ni_3_Fe-LDH – Dry AMO-Ni_3_Fe-borate-LDH powder (0.3 g) was dispersed in 20 mL of formamide. 10 mL of this mixture was then added dropwise to 100 mL of WS_2_ in dI-dC-water (0.27 mg mL^−1^) at a rate of 20 mL h^−1^ under a N_2_ atmosphere at 300 rpm. After 2 h, the mixture was centrifuged at 6000 rpm for 10 min, washed with formamide, and then ethanol, before drying in a vacuum oven at 65 °C.

### Materials characterisation

PXRD diffractograms were obtained using a Bruker D8 Advance Eco Diffractometer (2*θ* = 5–70°; Cu Kα, *λ*_α1_ = 1.54057 Å, *λ*_α2_ = 1.54433 Å, 40 kV, 25 mA). X-ray total scattering measurements were collected at the I15-1 beamline at the Diamond Light Source. LDH samples were loaded into 1 mm borosilicate capillaries and measured at room temperature. X-ray total scattering data were processed to a pair distribution function (PDF), *via* a sine Fourier transform and performed in GudrunX. XPS data were collected using a Thermo Scientific K-Alpha XPS instrument using a micro-focused monochromated Al Kα X-ray source at 1486.6 eV and 400 µm spot size. Charge neutralisation was applied using a combined low-energy/ion flood source. TGA was carried out using a PerkinElmer TGA 8000 under a continuous nitrogen flow from 30 to 800 °C at a ramp rate of 10 °C min^−1^. FTIR spectra were obtained using a Bruker VERTEX 80 FT-IR spectrometer fitted with a DuraSamplIR Diamond ATR. Transmittance was recorded at a resolution of 4 cm^−1^ over the range 4000–400 cm^−1^ for 128 scans. N_2_ adsorption–desorption isotherms were obtained for the powder samples using a Micromeritics Tristar II plus at –196 °C. Samples were degassed *ex situ* overnight at 115 °C prior to analysis. Specific surface areas were determined by the Brunauer–Emmett–Teller (BET) method. The pore size distributions were calculated using the built in Barrett–Joyner–Halenda (BJH) method from the N_2_ desorption isotherm. Scanning transmission electron microscopy (STEM) images were taken using a Zeiss Merlin scanning electron microscope with a 20–30 kV accelerating voltage and a working distance of 5 mm. An Agilent 5400 AFM was used to evaluate the sample thickness. Samples were prepared by drop-casting sample dispersions onto a Si wafer using a spin coater. Zeta potentials were recorded on samples dispersed in water (1 mg mL^−1^) using a Malvern Zetasizer Nano equipped with a 532 nm laser.

## Results and discussion

### Synthesis

Ni_3_Fe-LDH [Ni_0.75_Fe_0.25_(OH)_2_](A^*n*−^)_0.25/*n*_; A = borate ([B_3_O_3_(OH)_5_]^2−^ and [B(OH)_4_]^−^) were synthesised *via* the co-precipitation at a constant pH = 9 using metal nitrate precursors and B(OH)_3_, followed by aging for 24 h at 65 °C. Samples washed with water to neutral pH and dried *in vacuo* form ‘conventional’ agglomerated structures, and these are referred to as C-LDHs. AMO-LDHs were subjected to dispersion of the freshly prepared LDH ‘wet cake’ in isopropyl alcohol (IPA).^32^ TMD nanosheets were prepared *via* the reductive intercalation of Li on bulk TMD powders using *n*BuLi, before sonication in water to exfoliate the layers, yielding Li_*x*_MoS_2_ or Li_*x*_WS_2_ nanosheets.^27,33,34^ The TMD and LDH components were then combined by mixing the suspension of LDH in formamide with the dispersion of TMD in water, followed by isolation of the hybrid material powder by centrifugation.

### Structural characterisation

Atomic force microscopy (AFM) was used to determine the height profiles of the exfoliated TMD and LDH nanosheet materials. The AMO-Ni_3_Fe-borate-LDH material consists of particles with height profiles of 0.7–1.4 nm, increasing by a common factor of ∼0.7 nm (Fig. 3). These values align well with our previous work, in which we demonstrated that borate-LDHs can be exfoliated to well-dispersed, thin nanosheets through a facile AMOST strategy.^30^ The measured height profiles are also consistent with the theoretical thickness of a single LDH layer (0.48 nm), accounting for the additional height from the surface-adsorbed solvent layer of the particles.^35^ Analysis of the Li_*x*_MoS_2_ and Li_*x*_WS_2_ nanosheets shows they possess height profiles of 1.0–2.4 and 1.2–1.4 nm, respectively. These values are consistent with those previously reported for TMD single sheets synthesised *via* similar reductive intercalation methods, confirming exfoliation down to predominantly mono/few-layers.^26,27,36^ The hybrid materials, by contrast, show relatively larger height profiles. The MoS_2_/Ni_3_Fe-LDH material has a height profile range of 10.4–61.2 nm, whereas the WS_2_/Ni_3_Fe-LDH is 4.1–10.0 nm (Fig. S1). The lack of smaller-height features suggests that all the monolayer precursor materials have successfully assembled into larger particles.

**Fig. 3:**
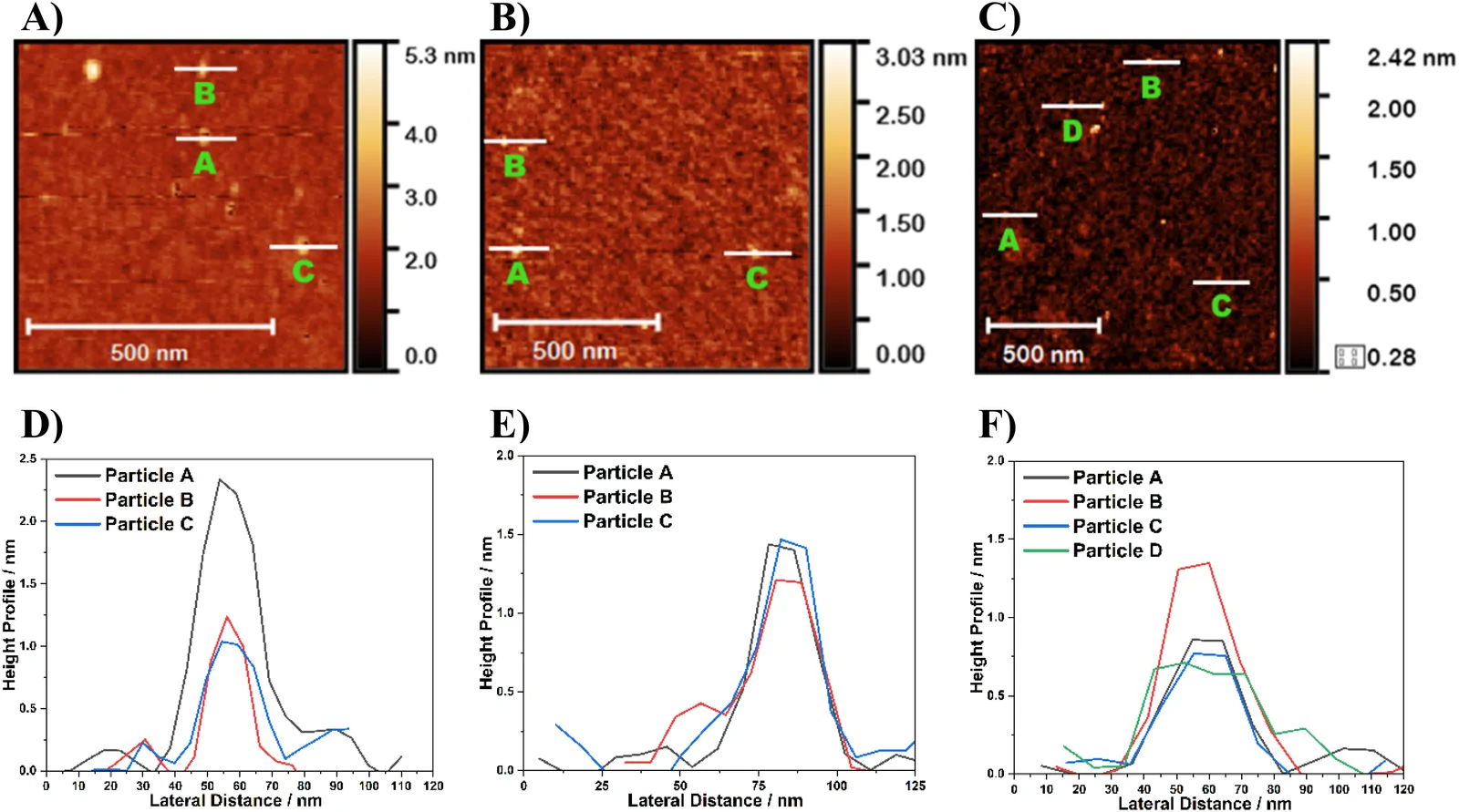
AFM images of the (A) Li_*x*_MoS_2_ (Nanosheet), (B) the Li_*x*_WS_2_ (Nanosheet), and (C) AMO-Ni_3_Fe-borate-LDH particles. The corresponding height profile cross-sections associated with each of the labelled particles (D), (E), and (F), respectively.

The electrostatic self-assembly of the TMD and LDH nanosheets is enabled by the opposite Zeta potentials (*ζ*) of the two materials. TMD nanosheets possess a *ζ*_TMD_ < 0 mV as a result of their formation *via* reductive intercalation of Li^+^ cations, and partial formation of M(III). LDHs, however, possess an intrinsic positive *ζ*_LDH_ > 0 mV. The sign of the Zeta potential indicates whether the suspended sheets will behave as a positively or negatively charged colloid, and the magnitude is a reflection of the colloidal stability.^37,38^ Table S1 shows the measured Zeta potentials for dispersions of the TMD and LDH, and for the hybrid materials. The data show that AMO-Ni_3_Fe-borate-LDH has a *ζ* = +36.5 mV, whereas the Li_*x*_MoS_2_ nanosheet sample has a *ζ* = −35.8 mV, which is almost equivalent in magnitude. Similarly, the Li_*x*_WS_2_ nanosheet sample has a *ζ* = –33.0 mV.

Powder X-ray diffraction (PXRD) data for the self-assembled hybrid materials, exfoliated TMD, and LDH precursors are shown in Fig. 4 and S2. Analysis of the PXRD patterns shows that there are Bragg reflections corresponding to the presence of a phase-pure rhombohedral (*R*3*m*) Ni_3_Fe-LDH crystalline phase for the C-Ni_3_Fe-borate-LDH and AMO-Ni_3_Fe-borate-LDH materials (JCPDS No. 40-0215). Fig. 4A shows the PXRD data for MoS_2_/Ni_3_Fe-LDH and its respective TMD and LDH precursors. Li_*x*_MoS_2_ nanosheet exhibits a phase transition from the 2H-polymorph (JCPDS No. 37-1492) of the bulk powder to the 1T-polymorph of MoS_2_, due to the reductive Li-intercalation and aqueous exfoliation steps (Fig. S2C). Fig. 4B shows the corresponding data for WS_2_/Ni_3_Fe-LDH, with the reference data for the 2H-polymorph of WS_2_ (JCPDS No. 84-1398). The data are similar to those of the MoS_2_/Ni_3_Fe-LDH analogue, except that no phase transition is observed for the Li_*x*_WS_2_ nanosheet sample (Fig. S2C).

**Fig. 4:**
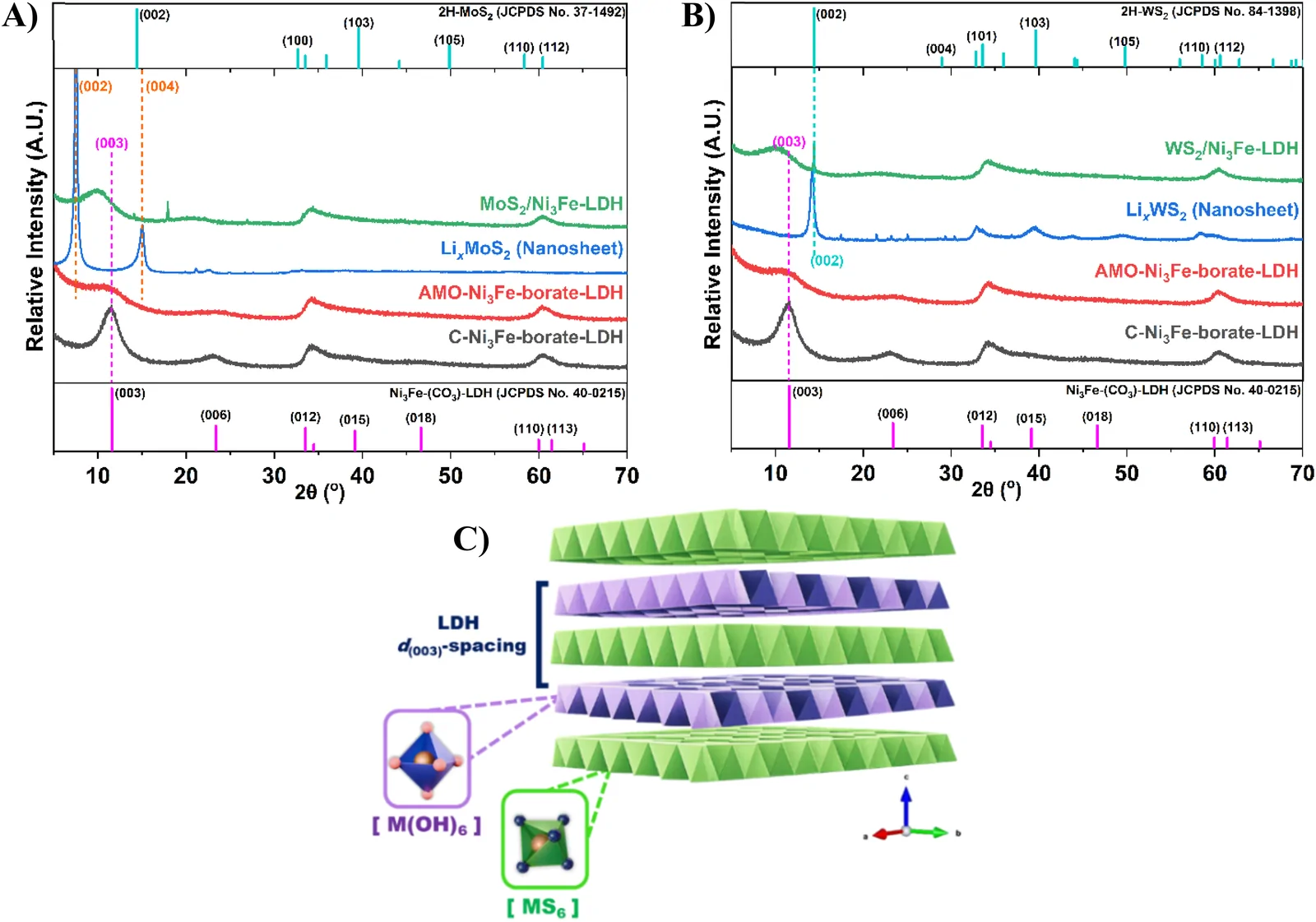
PXRD patterns for (A) the MoS_2_/Ni_3_Fe-LDH hybrid and corresponding TMD and LDH precursors – Li_*x*_MoS_2_ (Nanosheet) and AMO-Ni_3_Fe-borate-LDH, respectively, and (B) the corresponding data for the WS_2_/Ni_3_Fe-LDH hybrid. (C) Schematic structural model of the proposed stacking of TMD and LDH layers in the hybrid materials, showing the 2D TMD sheets (green) and the LDH sheets (purple). 002 and 004 Bragg reflections (orange) at 2*θ* = 7.7 and 15.2°, respectively, corresponding to the 1T-polymorph of MoS_2_.

Both hybrid materials, MoS_2_/Ni_3_Fe-LDH and WS_2_/Ni_3_Fe-LDH, possess a series of Bragg reflections consistent with an LDH phase, but the 00l Bragg harmonic series are shifted to lower 2*θ* values, suggesting a larger interlayer spacing. The calculated *d*_(003)_-spacing of MoS_2_/Ni_3_Fe-LDH is 8.86 Å, whereas WS_2_/Ni_3_Fe-LDH is 8.67 Å, both of which are ∼1 Å larger than the pristine LDH samples (Table 1). Previous work predicted the *d*_(003)_-spacing required to accommodate MoS_2_ sheets between LDH layers to range from 8.3–11.1 Å, with the range possibly reflecting different extents of reduction to the MoS_2_ layers,^26,27,39^ and therefore variations in the distortion of the TMD lattice.^40,41^

**Table 1:** Lattice parameters, using a rhombohedral (*R*3*m*) unit cell, for the C- and AMO-Ni_3_Fe-borate-LDH nanosheets and the hybrid materials MoS_2_/Ni_3_Fe-LDH and WS_2_/Ni_3_Fe-LDH. Ni_3_Fe-H_2_O denotes the simple water re-stacked material. CDL = crystallite domain length

Samples	*d*-Spacing/Å		Cell parameter/Å		CDL/nm	
	*d*_(003)_	*d*_(110)_	*c*	*a*	*D*_(003)_	*D*_(110)_
C-Ni_3_Fe-borate-LDH	7.75	1.54	23.24	3.08	5.70	11.13
AMO-Ni_3_Fe-borate-LDH	7.95	1.53	23.85	3.07	2.66	8.18
Ni_3_Fe-H_2_O	7.50	1.53	22.47	3.06	4.95	6.33
MoS_2_/Ni_3_Fe-LDH	8.86	1.53	26.57	3.07	4.15	7.46
WS_2_/Ni_3_Fe-LDH	8.67	1.53	26.00	3.07	3.74	7.46

The characteristic infrared (IR) vibrational bands of hydrotalcite-like compounds are present for all the LDH-derived materials: O–H stretches around 3350 cm^−1^, O–H bending modes at 1640 cm^−1^, CO_3_^2−^ stretches at 1360 cm^−1^, and M–O–M stretches around 720 cm^−1^ (Fig. S3). The MoS_2_/Ni_3_Fe-LDH shows additional stretching vibrations assigned to the MoS_2_ sheets, namely the *ν*(Mo=O), *ν*(S=O), and *ν*(Mo–S) at 950, 1113, and 624 cm^−1^.^39^ For the WS_2_/Ni_3_Fe-LDH, the vibrations of the W–S bending mode occur at ∼760 and 850 cm^−1^.^42^

Use of thermogravimetric analysis (TGA) provides information on the thermal properties of the synthesised materials. Shifts in the temperature of specific decomposition events can suggest differences in thermal stability, for example, due to alterations in the interlayer composition of the material. Notably, both the MoS_2_/Ni_3_Fe-LDH and WS_2_/Ni_3_Fe-LDH materials display an additional thermal decomposition feature at ∼255 °C, which is absent in both the pristine LDH and corresponding physical mixtures (Fig. S4). Dehydroxylation temperatures in LDHs are sensitive to the local chemical environment of the hydroxyl groups, including the identity and distribution of the layer cations and the interlayer environment.^43^ The emergence of this feature specifically upon formation of the hybrid materials is therefore consistent with modification of the local hydroxide environment following TMD/LDH assembly. However, TGA alone cannot uniquely assign the feature to dehydroxylation of the M–OH groups specifically at the TMD/LDH interface.

The materials were analysed using pair-distribution functions (XPDF), obtained from X-ray total scattering data, which gives information about the local environment of atoms in the structure. Total scattering data incorporates both Bragg and diffuse scattering, with the latter providing information on atom–atom correlations and deviations in local structure. The local structure of the Ni_3_Fe-borate-LDH is similar to that of previously synthesised Mg_3_Al-borate-LDH, with well-defined congruent maxima in the experimental *G*(*r*) profiles consistent with edge-sharing M(OH)_6_ octahedra (Fig. S5).^30,44^ Notably, the first and second shell M–M atom correlations in the Mg_3_Al-LDH at 3.03 and 5.20 Å, respectively, shift to higher *r*-values in the Ni_3_Fe-LDH, at 3.08 and 5.33 Å. In these mixed-metal LDHs, the M–M PDF features contain overlapping contributions from the different metal–metal pairs and are therefore best interpreted as average local M–M correlations, rather than individual pair distances. The observed shifts are consistent with changes in the average local metal–metal geometry associated with the change from Mg^2+^/Al^3+^ to Ni^2+^/Fe^3+^ cation compositions and their different ionic radii (*r*_+_: Ni^2+^ = 0.69 Å, Mg^2+^ = 0.72 Å, Fe^3+^ = 0.65 Å, Al^3+^ = 0.54 Å).^45^

In the hybrid materials, the characteristic atom–atom correlations from the Ni_3_Fe-LDH persist in both the MoS_2_/Ni_3_Fe-LDH and WS_2_/Ni_3_Fe-LDH (Fig. 5A). However, the M–OH correlation is at a shorter *r*-value (MoS_2_/Ni_3_Fe-LDH: 1.95 Å, WS_2_/Ni_3_Fe-LDH: 1.90 Å), suggesting a slight compression of the M(OH)_6_ octahedra present in the hybrid materials. The M–M (Ni/Fe–Ni/Fe) distance reflects the lattice *a*-parameter of the brucite-like layer and is consistent with the value determined from PXRD data (Table 1). An additional feature appears in both hybrid materials at ∼2.39 Å, corresponding to the first-shell peak of Mo–S and W–S bonds within the respective TMD sheets.^40,41^ The first-shell Mo–Mo and W–W correlations should appear at *r* ∼3.14 Å, but are partially obscured by the intense maximum originating from the LDH M–M correlation (Fig. 5A). Notably, additional features appear in both hybrid materials at *r* ∼3.95 and 4.46 Å, which are absent in the pristine Ni_3_Fe-LDH. These correlations are consistent with interfacial cross-layer interactions, such as Mo/W⋯Ni/Fe or S⋯Ni/Fe, highlighted in pink in Fig. 5A and B. However, contributions from higher-order correlations within the TMD components cannot be excluded. The features are therefore considered consistent with, rather than uniquely assigned to, possible interfacial correlations arising from the direct spatial proximity of TMD and LDH layers.^46^ Similarly, the medium-range features at 6.67 and 7.84 Å are consistent with higher-order interfacial correlations associated with the interstratified hybrid structure, although contributions from the constituent TMD structure cannot be excluded.^40^

**Fig. 5:**
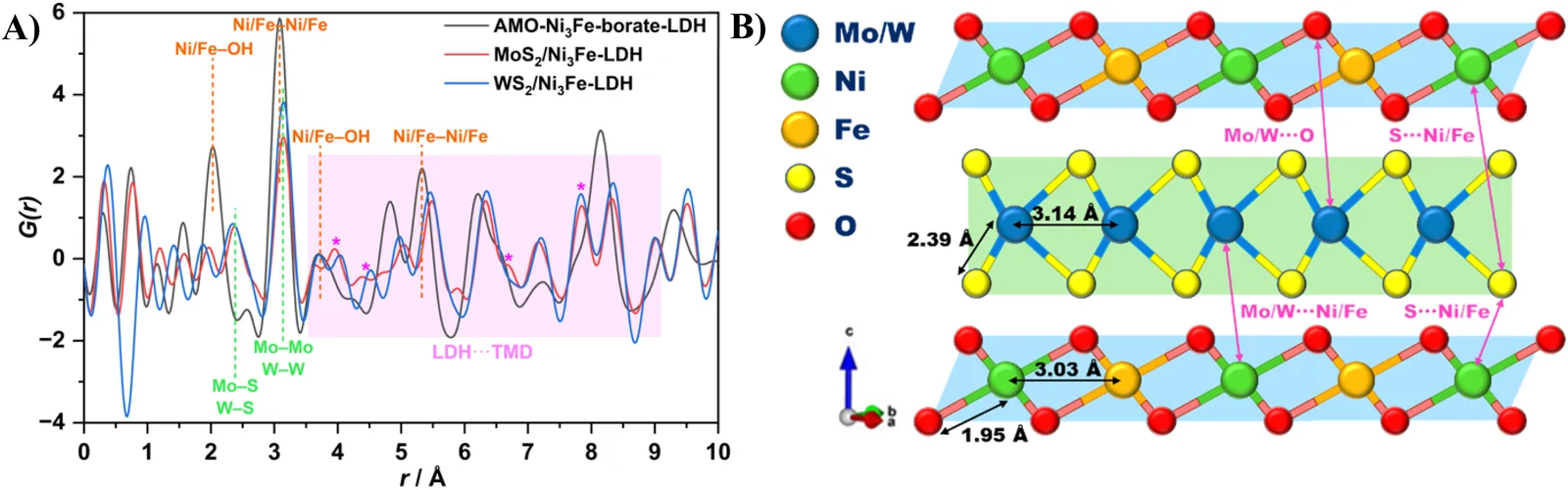
(A) X-ray pair distribution functions (XPDF) spectra for AMO-Ni_3_Fe-borate-LDH (black line), and the corresponding hybrid materials MoS_2_/Ni_3_Fe-LDH (red line) and WS_2_/Ni_3_Fe-LDH (blue line), and (B) diagram of NiFe-LDH and TMD layers highlighting the possible atom–atom correlations.

Brunauer–Emmett–Teller (BET) analysis of N_2_ adsorption–desorption isotherms provides information on the physically accessible surface area and pore structures in the synthesised hybrid materials (MoS_2_/Ni_3_Fe-LDH and WS_2_/Ni_3_Fe-LDH) and nanosheet precursors. Both the hybrid materials and nanosheet precursors display type IV isotherms, with a H3-type hysteresis loop, characteristic of platelets with slit-like pore geometries (Fig. S6).^47,48^ Barrett–Joyner–Halenda (BJH) analysis of the N_2_-desorption isotherm shows how the formation of the hybrid materials affects the pore size distribution, with retention of the small mesopores (20–100 Å) of the pristine LDH, but significant loss of large mesopores (100–500 Å) and macropores (>500 Å) (Fig. S7).^49^ The significant decrease in specific surface area (SSA) of the hybrid materials, relative to the AMO-Ni_3_Fe-LDH nanosheets, and the formation of small mesopore structures (20–40 Å), suggests the layers have re-stacked.

Angular dark-field scanning transmission electron microscopy (ADF-STEM) and EDX mapping were used to investigate the morphology and spatial distribution of the TMD- and LDH-derived elements within the hybrid materials. In ADF-STEM images, contrast intensity scales with atomic number (*Z*): brighter regions originate from heavier atoms (Mo, W) and dimmer regions from lighter atoms (Ni, Fe, O). The ADF-STEM data are consistent with the co-localisation of both high-*Z* (Mo, W) and lower-*Z* (Ni, Fe, O) components (Fig. 6). EDX elemental maps for the MoS_2_/Ni_3_Fe-LDH and WS_2_/Ni_3_Fe-LDH materials show co-localisation of Mo/W and S with Ni and Fe over the regions examined, with no clearly separated TMD- and LDH-rich domains observed at this length scale, consistent with spatial co-localisation of the constituent materials at this length scale. The spatial resolution of these measurements does not permit direct determination of the stacking sequence of individual TMD and LDH layers; the interstratified arrangement proposed in Fig. 4C is therefore a structural model supported by the combined characterisation rather than directly resolved by ADF-STEM.

**Fig. 6:**
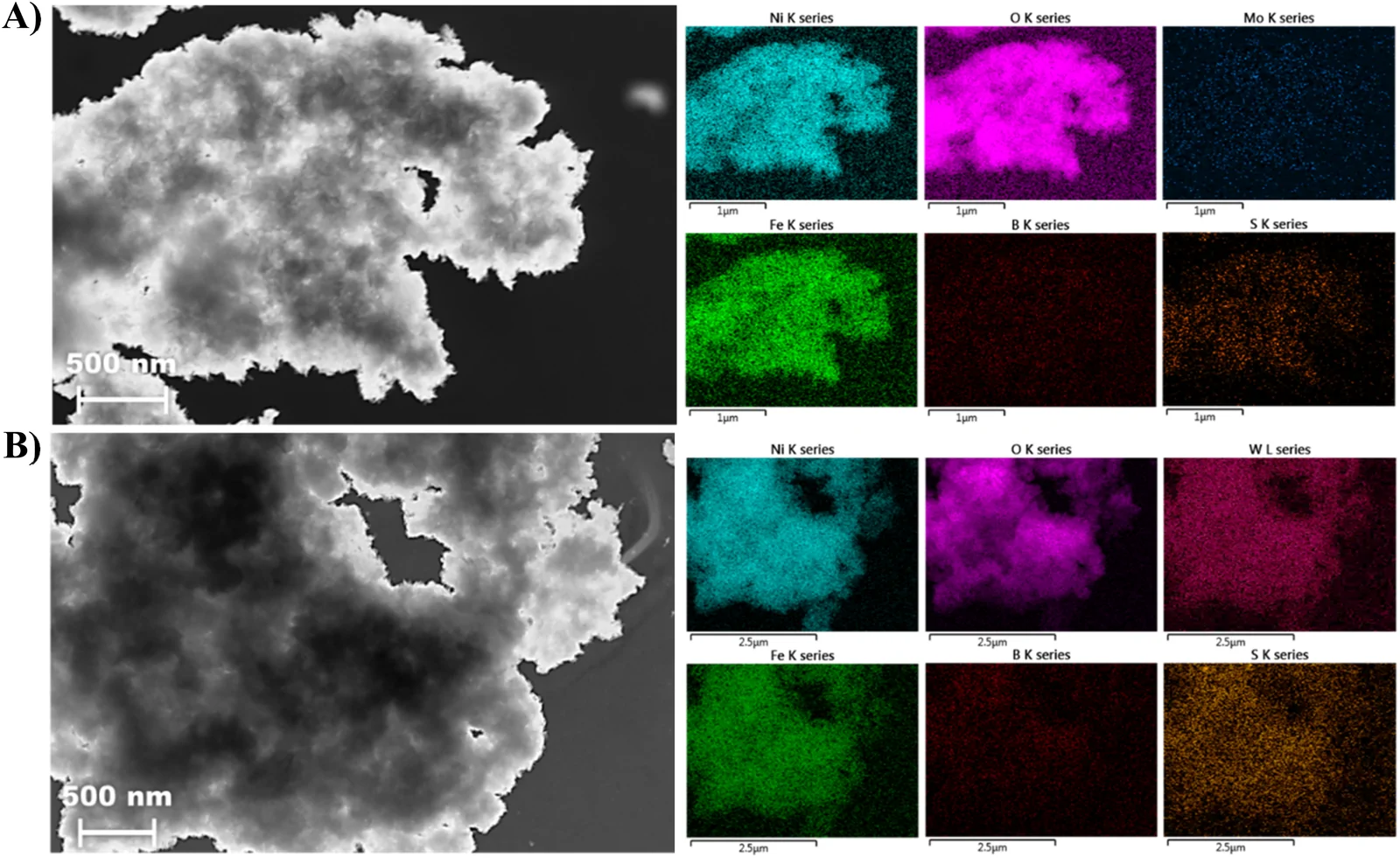
ADF-STEM images of (A) MoS_2_/Ni_3_Fe-LDH and (B) WS_2_/Ni_3_Fe-LDH hybrid materials and the corresponding energy dispersive X-ray elemental mapping data for Ni (cyan), Fe (green), O (pink), B (red), Mo (blue), W (magenta), and S (orange) in the sample.

X-ray photoelectron spectroscopy (XPS) was used to probe surface chemical states and changes in the local electronic environment upon formation of the TMD/LDH hybrid materials. The Ni 2p region of Ni (hydr)oxides is characterised by strong multiplet splitting and intense satellite features.^50^ For the pristine AMO-Ni_3_Fe-borate-LDH, the Ni 2p_3/2_ peak is centred around 855.8 eV, consistent with a Ni(OH)_2_-like environment (Fig. 7).^51^ The broad feature centred around ∼862 eV is a satellite peak arising primarily from ligand-to-metal charge-transfer (simultaneous excitation/shake-up) and multiplet effects associated with the interaction between the photoemission-induced Ni 2p hole and the partially occupied Ni 3d valence states. These satellites are therefore an intrinsic feature of the Ni 2p spectrum, rather than arising from additional Ni oxidation states.^52^

**Fig. 7:**
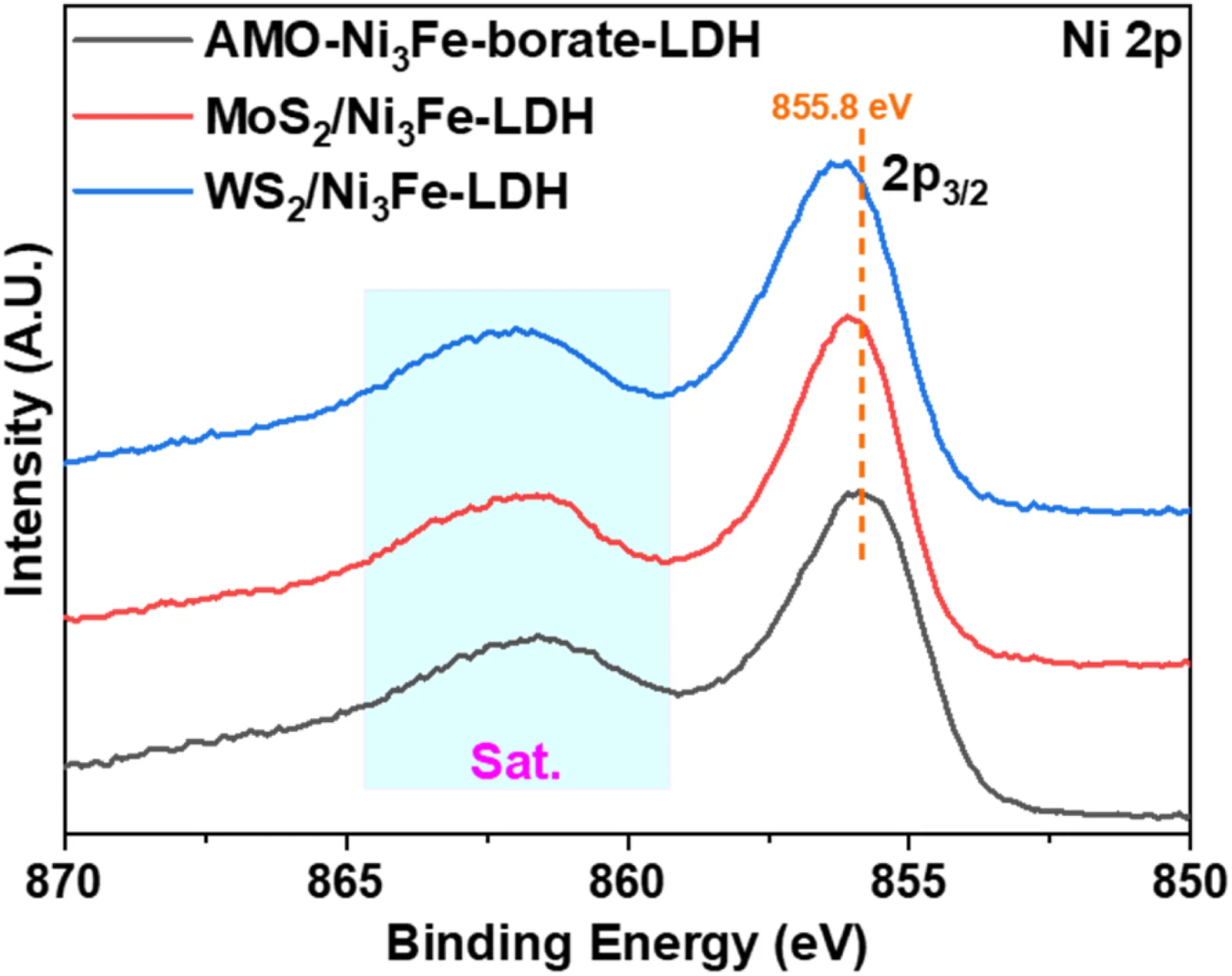
X-ray photoelectron spectroscopy (XPS) spectra of Ni 2p for AMO-Ni_3_Fe-borate-LDH (black line), and the corresponding hybrid materials MoS_2_/Ni_3_Fe-LDH (red line) and WS_2_/Ni_3_Fe-LDH (blue line). The satellite peak is highlighted by the cyan box (Sat).

In MoS_2_/Ni_3_Fe-LDH and WS_2_/Ni_3_Fe-LDH, the Ni 2p_3/2_ peak shifts to higher apparent binding energy relative to the pristine LDH; 856.1 and 856.3 eV, respectively. These shifts are consistent with modification of the local Ni electronic environment following formation of the hybrid material. Although partial oxidation towards a higher Ni valence cannot be excluded, the complex Ni 2p region alone does not uniquely establish a Ni^2+^ → Ni^3+^ conversion or provide a quantitative oxidation-state distribution, as changes in local coordination, charge redistribution and final-state effects may also contribute to the observed shifts.^53^ These observations are consistent with an electronic interaction between the TMD and LDH layers and may reflect charge redistribution across the interface. If charge redistribution does occur, the resulting increase in hole density may enhance the ability of the LDH layers to accept electrons from adsorbed intermediates (*OH, *O, *OOH) during the OER, which would improve activity.^25^ The larger shift observed for WS_2_/Ni_3_Fe-LDH may indicate a greater degree of electronic perturbation, which may contribute to differences in catalytic behaviour.

The emergence of additional cross-layer correlations around *r* ∼3.95 and 4.46 Å in the XPDF and the unique decomposition at ∼255 °C in the TGA provide the clearest evidence of interlayer coupling between the TMD and LDH. This is supported by nanoscale co-localisation of elements in the ADF-STEM-EDX images. These features are consistent with the formation of TMD/LDH hybrid materials rather than a simple physical mixture in both the MoS_2_/Ni_3_Fe-LDH and WS_2_/Ni_3_Fe-LDH materials.

### Measurement of OER performance

All prepared materials were tested for their electrocatalytic activity towards the OER using a 3-electrode setup (Fig. S9) in 1 M KOH at 25 °C, allowing comparison of the hybrid materials with their constituent nanosheet precursors and corresponding physical mixtures; full details of the electrochemical measurements are given in the SI. The AMO-Ni_3_Fe-borate-LDH shows an overpotential of 327 mV at 10 mA cm^−2^ (*η*_10_), as expected for pristine Ni_*x*_Fe-LDHs.^15,54^ The overpotentials of both MoS_2_/Ni_3_Fe-LDH and WS_2_/Ni_3_Fe-LDH decrease by over 70 mV, compared to the pristine Ni_3_Fe-LDH, which is already regarded as a highly active OER electrocatalyst (Fig. 8A and B). MoS_2_/Ni_3_Fe-LDH and WS_2_/Ni_3_Fe-LDH exhibit low overpotentials of 258 and 248 mV, respectively, with small Tafel slopes of 49 and 37 mV dec^−1^, comparable to previously reported hybrid materials (*η*_10_ ∼ 210–250 mV; Fig. 8).^26,27^ This decrease in overpotential for the hybrid materials thus represents a significant increase in activity relative to the pristine LDH. To confirm that intimate mixing of the TMD and LDH components is essential for maximising electrocatalytic performance, physical mixtures of MoS_2_/WS_2_ and Ni_3_Fe-LDH were tested, yielding overpotentials of *η*_10_ = 296 mV and *η*_10_ = 279 mV, respectively (Fig. S11). The superior OER performance of the hybrid materials relative to the physical mixtures is consistent with beneficial electronic interaction between the LDH and TMD components that is promoted by the interstratified architecture,^55^ aligning with previously reported density functional theory (DFT) calculations.^27^

**Fig. 8:**
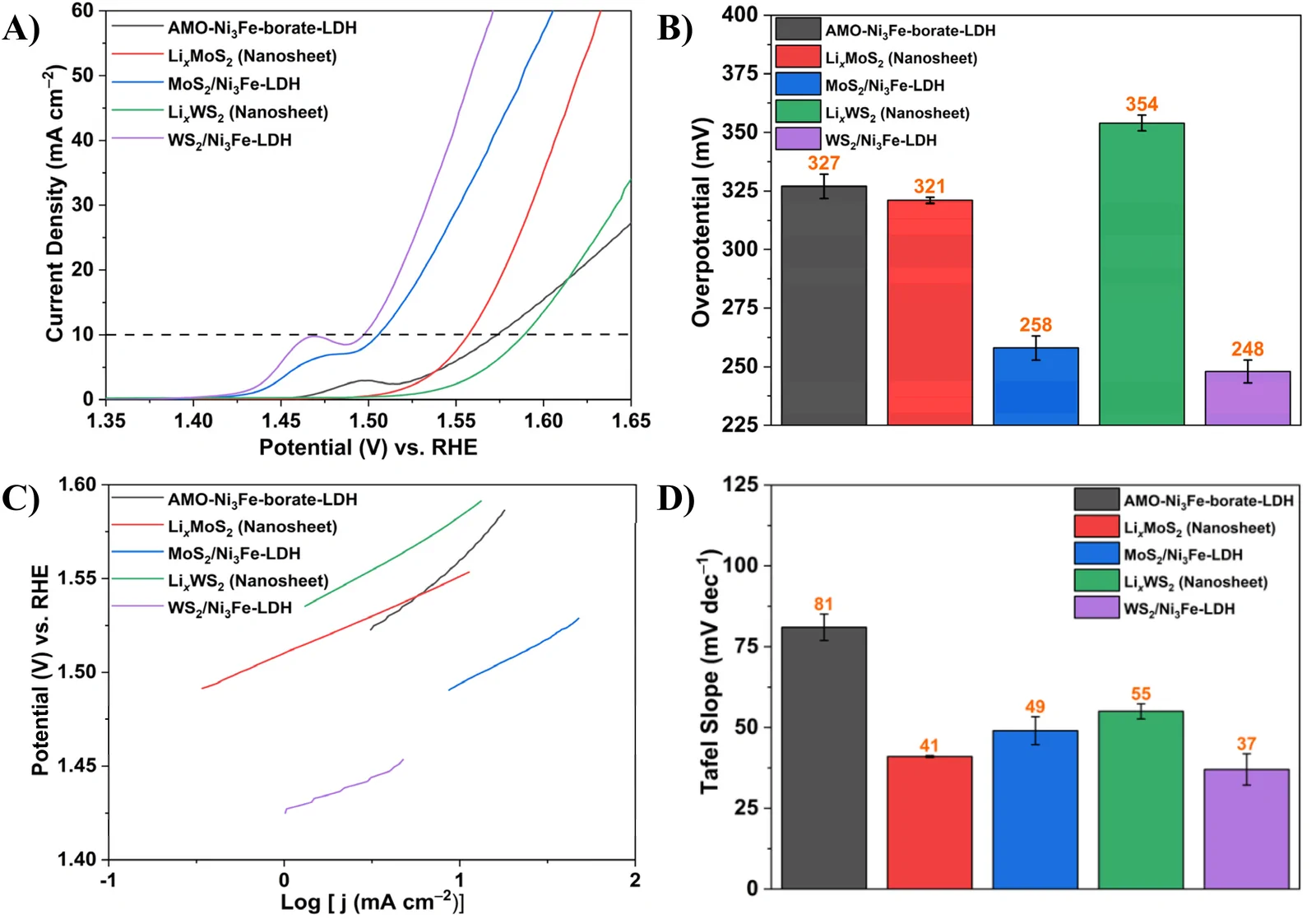
(A) Linear sweep voltammetry (LSV) curves, and (B) extracted overpotential at a current density of 10 mA cm^−2^, (C) Tafel plots for OER, and (D) Tafel slope extracted from the gradient of the Tafel plots for; the MoS_2_/Ni_3_Fe-LDH and WS_2_/Ni_3_Fe-LDH hybrid materials, and the corresponding TMD and LDH nanosheet precursors – AMO-Ni_3_Fe-borate-LDH, Li_*x*_MoS_2_ (Nanosheet), and Li_*x*_WS_2_ (Nanosheet).

The Tafel slopes of MoS_2_/Ni_3_Fe-LDH and WS_2_/Ni_3_Fe-LDH are comparable to the Li_*x*_MoS_2_ and Li_*x*_WS_2_ nanosheet materials alone, yet significantly lower than the AMO-Ni_3_Fe-borate-LDH, indicating superior OER kinetics for the TMD-based materials compared to the LDH (Fig. 8C and D). This is further confirmed by the Nyquist plots from electrochemical impedance spectroscopy (EIS) measurements (Fig. 9A and S12). Both MoS_2_/Ni_3_Fe-LDH and WS_2_/Ni_3_Fe-LDH exhibit semi-circles of smaller diameter than AMO-Ni_3_Fe-borate-LDH, which implies reduced charge-transfer resistance.^56^ EIS data were fitted using equivalent circuit models to extract the solution resistance (*R*_s_), interfacial resistance (*R*_f_), and charge-transfer resistance (*R*_ct_) (Fig. 9A insert).^56,57^ The measured *R*_ct_ at 1.525 V (*vs.* V_RHE_) for MoS_2_/Ni_3_Fe-LDH and WS_2_/Ni_3_Fe-LDH equates to 11.1 and 10.6 W, respectively, significantly smaller than the *R*_ct_ of pristine LDH (52.0 W) and TMD nanosheets (Li_*x*_MoS_2_ = 143.3 W; Li_*x*_WS_2_ = 421.7 W) and values reported for similar materials (Table S2 and S3).^26,27^ The low *R*_ct_ values for MoS_2_/Ni_3_Fe-LDH and WS_2_/Ni_3_Fe-LDH are consistent with intimate interfacial contact between the LDH and TMD sheets and more favourable charge-transfer within the hybrid architecture. This suggests that interstratification promotes more favourable charge-transfer within the TMD/LDH hybrid material relative to the pristine LDH, contributing to the higher OER activity observed.^38,58^

**Fig. 9:**
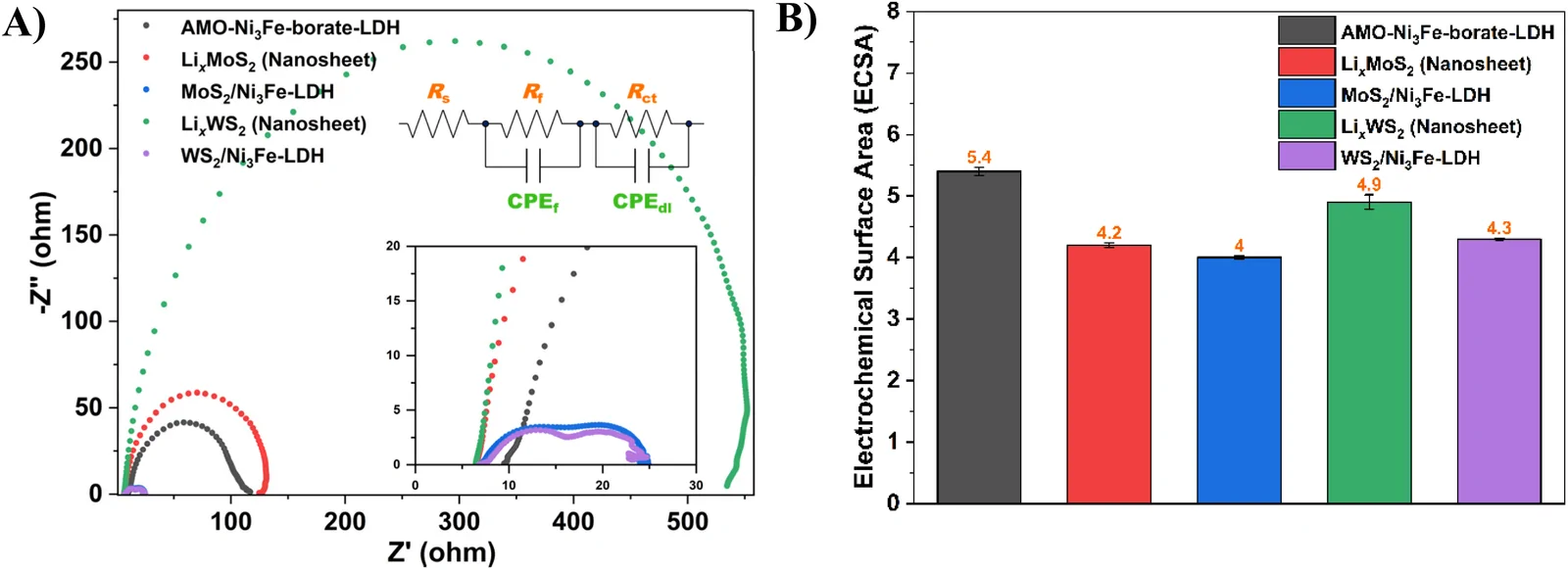
(A) Electrochemical impedance spectroscopy (EIS) Nyquist plots, (B) electrochemical surface area (ECSA) measured from cyclic voltammetry (CV) scans at scan rates of 5, 10, 20, 50, 100, and 200 mV s^−1^ for; the MoS_2_/Ni_3_Fe-LDH and WS_2_/Ni_3_Fe-LDH hybrid materials, and the corresponding TMD and LDH nanosheet precursors – AMO-Ni_3_Fe-borate-LDH, Li_*x*_MoS_2_ (Nanosheet), and Li_*x*_WS_2_ (Nanosheet).

The influence of the TMD sheet on interfacial charge-transfer between the LDH and TMD layers can be further understood from the shift in the redox peaks during voltammetry measurements. Islam *et al.* have previously suggested that the interstratified MoS_2_ nanosheet induces a shift in this Ni redox peak to a lower potential, due to possible interfacial charge-transfer between the TMD and LDH sheets in the hybrid material.^27^ The Ni^2+^ → Ni^3+^ redox peak at 1.498 V in AMO-Ni_3_Fe-borate-LDH shifts to a lower potential for both hybrid materials: 1.477 V for MoS_2_/Ni_3_Fe-LDH, and 1.468 V for WS_2_/Ni_3_Fe-LDH (Fig. S13). Interfacial charge-transfer may lead to electronic redistribution across the heterointerface, which can modify the electronic structure of the Ni–Fe catalytic centres and influence the subsequent formation of the catalytically active oxyhydroxide phase under OER conditions.^58,59^ For the WS_2_/Ni_3_Fe-LDH, the shift in the Ni redox peak is slightly greater and consistent with XPS results (Fig. 7), suggesting WS_2_ exerts a larger electronic perturbation on the Ni_3_Fe-LDH pre-catalyst than MoS_2_. The absence of a comparable shift in the Ni redox peak in the physical mixtures indicates that formation of the hybrid architecture modifies the Ni-centred redox environment. Together with the XPS and EIS observations, this is consistent with interfacial electronic coupling, suggesting that the interstratified architecture is important for the observed electrochemical perturbation, rather than just the proximity of the materials.

The electrochemically active surface area (ECSA) of the synthesised hybrid materials was estimated from their double-layer capacitance (*C*_dl_), calculated from cyclic voltammetry (CV) scans in a non-faradaic region (0.975–1.125 V *vs.* V_RHE_) over multiple scan rates (5, 10, 20, 50, 100, 200 mV s^−1^; Fig. S14 and S15). Determination of the (ECSA = *C*_dl_/*C*_s_; specific capacitance, *C*_s_, of 40 µF cm^−2^ (for metal surfaces in alkaline media)) provides an approximate comparative estimate of the electrochemically accessible surface area, which may correlate with the number of accessible catalytic centres.^60^ ECSA can differ distinctly from both the physical surface area of the catalyst (from BET analysis) and the geometric surface of the glassy-carbon working electrode, and is essential for distinguishing the intrinsic catalytic activity of the material from surface area effects. The MoS_2_/Ni_3_Fe-LDH and WS_2_/Ni_3_Fe-LDH materials possess an ECSA = 4.0 and 4.3, respectively, which are markedly lower than that of the pristine LDH (ECSA = 5.43; Fig. 9B). This reduction in ECSA suggests that the interstratification of the TMD leads to a decreased electrochemically accessible surface area, consistent with the decreased BET-derived specific surface area, and a consequence of the re-stacking of LDH layers upon forming the hybrid materials. Thus, for the same catalyst loading, the hybrid materials display improved apparent OER performance; after normalisation by ECSA, the data still suggest a beneficial contribution from the interfacial electronic coupling (Fig. S16). The sustained improvements for both hybrid materials in the ECSA-normalised data highlight that interstratification contributes to the observed performance enhancement, providing evidence that electronic coupling between TMD and LDH layers influences the electrocatalytic behaviour of the Ni–Fe pre-catalysts.

To assess the electrocatalytic stability of the synthesised materials, chronopotentiometry (CP) was performed over 12 h at a fixed current density of 10 mA cm^−2^ (Fig. 10A and S17A). The drift rate, determined from the rate of change in potential required to maintain the current density of 10 mA cm^−2^, is lower for MoS_2_/Ni_3_Fe-LDH (Drift rate = 1.23 mV h^−1^) and WS_2_/Ni_3_Fe-LDH (Drift rate = 1.05 mV h^−1^), compared to pristine LDH (Drift rate = 3.23 mV h^−1^; Fig. 10B and S17B). The lower drift rate indicates the improved stability of the hybrid materials over the 12 h test period. These measurements provide a comparative assessment of durability under the present three-electrode conditions rather than a demonstration of long-term practical electrolyser stability. The 12 h measurements shown in Fig. 10 and S17, and the extended measurements shown in Fig. S19 were obtained from independently prepared electrodes and represent separate experimental runs. In the extended experiments, MoS_2_/Ni_3_Fe-LDH and WS_2_/Ni_3_Fe-LDH maintained operation at 10 mA cm^−2^ current density for 19.1 and 21.6 h, respectively, before loss of stable operation (Fig. S19). These separate extended measurements provide additional information on durability under the steady-state conditions studied, although substantially longer testing would be required to establish long-term practical stability.

**Fig. 10:**
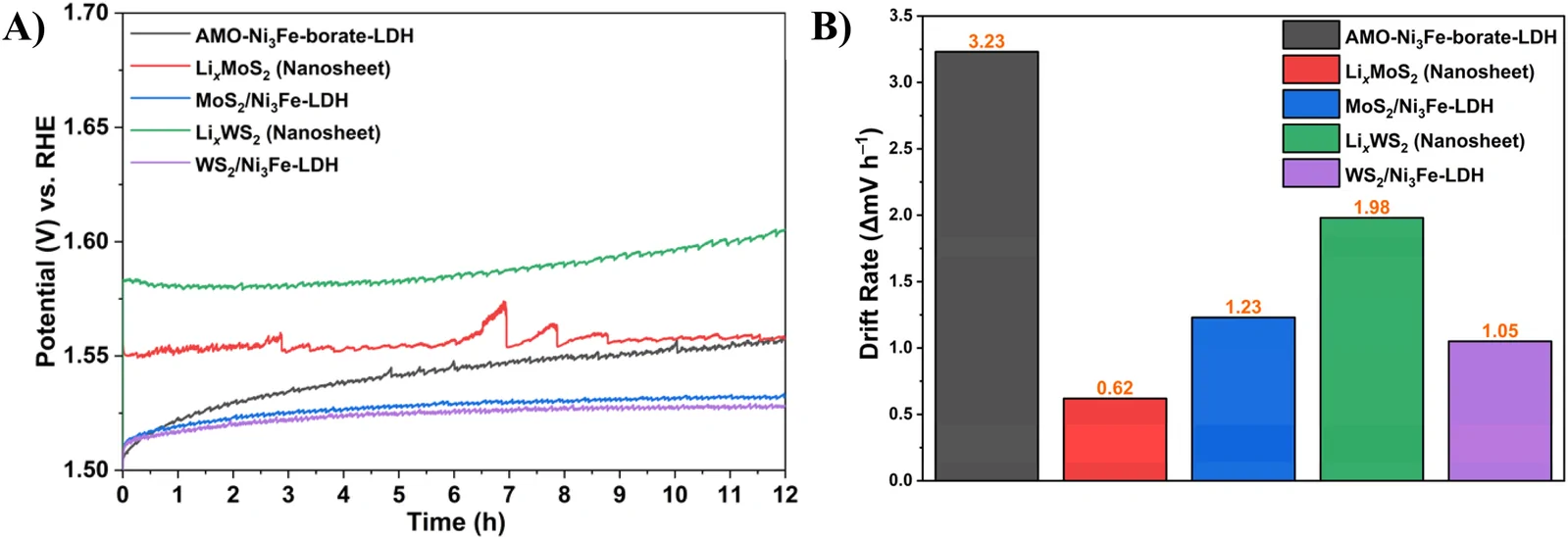
(A) Chronopotentiometry (CP) data at a current density of 10 mA cm^−2^ for 12 h, and (B) the average potential drift rate measured from the gradient between *t* = 0 and *t* = 12 h for; the MoS_2_/Ni_3_Fe-LDH and WS_2_/Ni_3_Fe-LDH hybrid materials, and the corresponding TMD and LDH nanosheet precursors – AMO-Ni_3_Fe-borate-LDH, Li_*x*_MoS_2_ (Nanosheet), and Li_*x*_WS_2_ (Nanosheet).

Accelerated durability testing (ADT) was also performed over 500 CV cycles, as a complementary assessment of electrochemical stability under dynamic OER conditions (1.125–1.625 V *vs.* V_RHE_; Fig. S20). Unlike CP, which probes catalyst stability under sustained operation at a constant current density, ADT repeatedly cycles the catalyst through oxidative and reductive potentials. ADT therefore provides a more stringent assessment of the catalyst's resistance to repeated redox-induced structural and electrochemical changes. Together, CP and ADT therefore probe complementary aspects of catalyst durability.

Both the pristine LDH and MoS_2_/WS_2_ nanosheets show significant variation between cycles 1 and 500, characterised by the changes in the shape and position of the CV curves. In contrast, the MoS_2_/Ni_3_Fe-LDH and WS_2_/Ni_3_Fe-LDH hybrid materials exhibited minimal change, with cycles 1 and 500 retaining similar shapes and positions. The limited evolution of the CV curves is consistent with greater preservation of the electrochemical response during repeated redox cycling relative to the pristine LDH and TMD materials. Furthermore, the comparatively stable CV profiles suggest that the hybrid materials are less susceptible to degradation under repeated oxidative and reductive cycling, consistent with enhanced durability of the interstratified pre-catalysts under OER cycling conditions.

## Conclusion

The hybrid materials MoS_2_/Ni_3_Fe-LDH and WS_2_/Ni_3_Fe-LDH, consisting of interstratified TMD and LDH monolayers, show high OER electrocatalytic activity. The enhanced performance is consistent with electronic coupling across the TMD/LDH heterointerface, which appears to lower charge-transfer resistance and may promote more favourable interfacial charge transport in the hybrid pre-catalysts under OER conditions.^61^ They also show significantly reduced overpotentials and Tafel slopes relative to either the pristine monolayer materials or physical mixtures of the TMD and LDH, indicating that the interstratification enhances OER activity. To the best of our knowledge, this is the first report of a WS_2_/Ni_3_Fe-LDH hybrid, which demonstrates improved OER performance compared to the MoS_2_/Ni_3_Fe-LDH analogue, with greater electronic perturbation of the Ni sites in the LDH layers, as indicated by XPS and voltammetry data. Additionally, the hybrid materials exhibit improved electrochemical stability towards the OER under the conditions studied, showing lower potential drift than the pristine LDH during 12 h CP measurements. Separate extended CP measurements maintained an operating current density of 10 mA cm^−2^ for ∼20 h; however, substantially longer testing would be required to establish long-term practical stability. Both hybrids also show minimal changes in *η*_10_ following accelerated durability testing over 500 CV cycles. This work demonstrates that controlled LDH nanosheet re-stacking can be used to create hybrid pre-catalysts with improved OER behaviour. Future research aims to explore the use of other 2D layered materials to access a wide array of hybrid materials for alternative photo- and electrocatalytic applications.

## Author contributions

M. R. L. W. and D. O. H. conceived the original idea. M. R. L. W. carried out the synthesis, characterisation, and data analysis of the materials. M. R. L. W. wrote the manuscript with input from all authors.

## Conflicts of interest

There are no conflicts to declare.

## Supplementary Material

RA-016-D6RA07498G-s001

## Data Availability

The data supporting this article have been included as part of the supplementary information (SI). Supplementary information: additional structural, morphological, thermal and surface characterisation, detailed electrochemical measurements and analysis, durability testing, and comparison with previously reported 2D-material/LDH OER electrocatalysts. See DOI: https://doi.org/10.1039/d6ra07498g.
