# Supplementary material for: Oxygen evolution catalysts by self-assembly of MS2 (M = Mo, W) and Ni3Fe-LDH nanosheets
Source: RSC Adv. 2026 Oct 7;16(46):60740–51. doi: 10.1039/d6ra07498g (PMC13644399; doi:10.1039/d6ra07498g)
Supplement: Supplementary file 1 [file RA-016-D6RA07498G-s001.pdf]

Supplementary Information (SI) for RSC Advances.
This journal is © The Royal Society of Chemistry

Supplementary Information

Oxygen evolution catalysts by self-assembly of MS_2 (M = Mo, W) and Ni_3Fe-LDH nanosheets

Matthew R. L. Williams,[a] Yang Li,[a] Roland C. Turnell-Ritson,[a] Dermot O'Hare,[a]

[a]Chemistry Research Laboratory, *Department of Chemistry, University of Oxford, Mansfield Road, Oxford, OX1 3TA UK*

Figure S1 – AFM images of **A)** the MoS_2/Ni_3Fe-LDH hybrid and **B)** the WS_2/Ni_3Fe-LDH hybrid and the corresponding height profile cross-sections associated with each of the labelled particles; **C)** and **D)**. 7

Figure S2 – PXRD patterns for **A)** the MoS_2/Ni_3Fe-LDH hybrid (green line) and corresponding TMD and LDH precursors – Li_xMoS_2 (Nanosheet) (gold line) and AMO-Ni_3Fe-borate-LDH (red line), respectively, and **B)** the corresponding data for the WS_2/Ni_3Fe-LDH hybrid. "(PM)" denotes the physical mixture of TMD and LDH, and Ni_3Fe-H_2O denotes the simple water re-stacked material (blue line). **C)** PXRD patterns for the untreated, bulk TMD powders and the corresponding Li_xMS_2 (Nanosheet) materials. 8

Figure S3 – FTIR spectra for **A)** the MoS_2/Ni_3Fe-LDH hybrid (green line) and corresponding TMD and LDH precursors – Li_xMoS_2 (Nanosheet) (gold line) and AMO-Ni_3Fe-borate-LDH (red line), respectively, and **B)** the corresponding data for the WS_2/Ni_3Fe-LDH hybrid. "(PM)" denotes the physical mixture of TMD and LDH, and Ni_3Fe-H_2O denotes the simple water re-stacked material (blue line). The region denoted by * is an artefact of the sample stage. 9

Figure S4 – TGA curves for **A)** the MoS_2/Ni_3Fe-LDH hybrid (green line) and corresponding TMD and LDH precursors – Li_xMoS_2 (Nanosheet) (gold line) and AMO-Ni_3Fe-borate-LDH (red line), respectively, and **B)** the corresponding data for the WS_2/Ni_3Fe-LDH hybrid. "(PM)" denotes the physical mixture of TMD and LDH, and Ni_3Fe-H_2O denotes the simple water re-stacked material (blue line). TGA curves were collected under N_2 at a ramp rate of 10 °C min^{-1} with the accompanying differential TGA (dTGA) curves shown as dotted lines. 10

Figure S5 – X-ray pair distribution functions (XPDF) spectra for **A)** Mg_3Al-borate-LDH (black = C-LDH, red = AMO-LDH) and Ni_3Fe-borate-LDHs (blue = C-LDH, green = AMO-LDH) and **B)** AMO-Ni_3Fe-borate-LDH (black line), and the corresponding hybrid materials MoS_2/Ni_3Fe-LDH (red line) and WS_2/Ni_3Fe-LDH (blue line). 11

Figure S6 – A) N_2 adsorption-desorption for the MoS_2/Ni_3Fe-LDH hybrid and corresponding TMD and LDH precursors – Li_xMoS_2 (Nanosheet) and AMO-Ni_3Fe-borate-LDH, respectively, and the corresponding data for the WS_2/Ni_3Fe-LDH hybrid. "(PM)" denotes the physical mixture of TMD and LDH, and Ni_3Fe-H_2O denotes the simple water re-stacked material. **B)** Magnified version of **A)** in the y-axis. .... **Error! Bookmark not defined.**

Figure S7 – Summary of surface analysis data for the MoS_2/Ni_3Fe-LDH hybrid and corresponding TMD and LDH precursors – Li_xMoS_2 (Nanosheet) and AMO-Ni_3Fe-borate-LDH, respectively, and the corresponding data for the WS_2/Ni_3Fe-LDH hybrid. "(PM)" denotes the physical mixture of TMD and LDH, and Ni_3Fe-H_2O denotes the simple water re-stacked material. **A)** BET-derived specific surface areas and total pore volumes, and **B)** BJH desorption-derived pore size distributions. 12

Figure S8 – X-ray photoelectron spectroscopy (XPS) spectra of **A)** Fe 2p and **B)** O 1s for AMO-Ni_3Fe-borate-LDH (black line), and the corresponding hybrid materials MoS_2/Ni_3Fe-LDH (red line) and WS_2/Ni_3Fe-LDH (blue line). XPS survey spectra for **C)** AMO-Ni_3Fe-borate-LDH, **D)** MoS_2/Ni_3Fe-LDH, and **E)** WS_2/Ni_3Fe-LDH. 13

Figure S9 – Experimental 3-electrode setup consisting of Hg/HgO reference electrode (RE), 1 cm^2 Pt counter electrode (CE), and a glassy-carbon working electrode (WE) immersed in 1 M KOH at 25 °C. 14

Figure S10 – Cyclic voltammetry (CV) data for; **A)**, **B)** "C-Ni_3Fe-A-LDH"; A = carbonate or borate, denotes the conventionally synthesised LDH, **C)** AMO-Ni_3Fe-borate-LDH, **D)**, **G)** Li_xMoS_2 (Nanosheet) and Li_xWS_2 (Nanosheet), **E)**, **H)** the MoS_2/Ni_3Fe-LDH and WS_2/Ni_3Fe-LDH hybrid materials, and **F)**, **I)** the corresponding physical mixtures of TMD and LDH denoted "(PM)". CV data were recorded at a scan rate of 100 mV s^{-1} in the range: 0.0 – 0.7 V vs Hg/HgO reference electrode in 1 M KOH. 15

Figure S11 – **A)** Linear sweep voltammetry (LSV) curves, and **B)** extracted overpotential at a current density of 10 mA cm^{-2}, **C)** Tafel plots for OER, and **D)** Tafel slope extracted from the gradient of the Tafel plots for; the MoS_2/Ni_3Fe-LDH and WS_2/Ni_3Fe-LDH hybrid materials, and the corresponding TMD and LDH nanosheet precursors – AMO-Ni_3Fe-borate-LDH, Li_xMoS_2 (Nanosheet), and Li_xWS_2 (Nanosheet). "C-Ni_3Fe-A-LDH"; A = carbonate or borate, denotes the conventionally synthesised LDH and "(PM)" denotes the physical mixture of TMD and LDH. 16

Figure S12 – Electrochemical impedance spectroscopy (EIS) Nyquist plots for; the MoS_2/Ni_3Fe-LDH and WS_2/Ni_3Fe-LDH hybrid materials, and the corresponding TMD and LDH nanosheet precursors – AMO-Ni_3Fe-borate-LDH, Li_xMoS_2 (Nanosheet), and Li_xWS_2 (Nanosheet). "C-Ni_3Fe-A-LDH"; A = carbonate or borate, denotes the conventionally synthesised LDH and "(PM)" denotes the physical mixture of TMD and LDH. 17

Figure S13 – LSV data for the MoS_2/Ni_3Fe-LDH and WS_2/Ni_3Fe-LDH hybrid materials, and the corresponding AMO-Ni_3Fe-borate-LDH measured in 1 M KOH, highlighting the potential of the Ni redox peak prior to the OER onset potential. 18

Figure S14 – Cyclic voltammetry (CV) scans at scan rates of 5, 10, 20, 50, 100, and 200 mV s^{-1} for; **A)**, **B)** "C-Ni_3Fe-A-LDH"; A = carbonate or borate, denotes the conventionally synthesised LDH, **C)** AMO-Ni_3Fe-borate-LDH, **D)**, **G)** Li_xMoS_2 (Nanosheet) and Li_xWS_2 (Nanosheet), **E)**, **H)** the MoS_2/Ni_3Fe-LDH and WS_2/Ni_3Fe-LDH hybrid materials, and **F)**, **I)** the corresponding physical mixtures of TMD and LDH denoted "(PM)". 19

Figure S15 – **A)** Plot of capacitive current, j_{capa}, against scan rate, and **B)** electrochemical surface area (ECSA) measured from cyclic voltammetry (CV) scans at scan rates of 5, 10, 20,

50, 100, and 200 mV s^{-1} for; the MoS_2/Ni_3Fe-LDH and WS_2/Ni_3Fe-LDH hybrid materials, and the corresponding TMD and LDH nanosheet precursors – AMO-Ni_3Fe-borate-LDH, Li_xMoS_2 (Nanosheet), and Li_xWS_2 (Nanosheet). "C-Ni_3Fe-A-LDH"; A = carbonate or borate, denotes the conventionally synthesised LDH and "(PM)" denotes the physical mixture of TMD and LDH. 20

Figure S16 – LSV data normalised by dividing by the ECSA for; the MoS_2/Ni_3Fe-LDH and WS_2/Ni_3Fe-LDH hybrid materials, and the corresponding TMD and LDH nanosheet precursors – AMO-Ni_3Fe-borate-LDH, Li_xMoS_2 (Nanosheet), and Li_xWS_2 (Nanosheet). "C-Ni_3Fe-A-LDH"; A = carbonate or borate, denotes the conventionally synthesised LDH and "(PM)" denotes the physical mixture of TMD and LDH. 20

Figure S17 – A) Chronopotentiometry (CP) data at a current density of 10 mA cm^{-2} for 12 h, and **B)** the average potential drift rate measured from the gradient between t = 0 and t = 12; the MoS_2/Ni_3Fe-LDH and WS_2/Ni_3Fe-LDH hybrid materials, and the corresponding TMD and LDH nanosheet precursors – AMO-Ni_3Fe-borate-LDH, Li_xMoS_2 (Nanosheet), and Li_xWS_2 (Nanosheet). "C-Ni_3Fe-A-LDH"; A = carbonate or borate, denotes the conventionally synthesised LDH and "(PM)" denotes the physical mixture of TMD and LDH. 21

Figure S18 – LSV measured before "[Pre]" and after "[Post]" 12 h CP experiments at 10 mA cm^{-2} for **A)** the MoS_2/Ni_3Fe-LDH hybrid and corresponding TMD and LDH precursors – Li_xMoS_2 (Nanosheet) and AMO-Ni_3Fe-borate-LDH, respectively, and **B)** the corresponding data for the WS_2/Ni_3Fe-LDH hybrid. "(PM)" denotes the physical mixture of TMD and LDH, and Ni_3Fe-H_2O denotes the simple water re-stacked material. 22

Figure S19 – Chronopotentiometry (CP) data at a current density of 10 mA cm^{-2}, for the MoS_2/Ni_3Fe-LDH and WS_2/Ni_3Fe-LDH hybrid materials, measured in independent extended experiments until loss of stable operation. These measurements were performed using separately prepared electrodes and are independent of the 12 h CP measurements shown in Fig. 10 and Fig. S17. 23

Figure S20 – Accelerated durability testing (ADT) data measured using cyclic voltammetry (CV) for; **A)**, **B)** "C-Ni_3Fe-A-LDH"; A = carbonate or borate, denotes the conventionally synthesised LDH, **C)** AMO-Ni_3Fe-borate-LDH, **D)**, **F)** the MoS_2/Ni_3Fe-LDH and WS_2/Ni_3Fe-LDH hybrid materials, and **E)**, **G)** the corresponding physical mixtures of TMD and LDH denoted "(PM)". CV data were recorded at a scan rate of 100 mV s^{-1} in the range: 0.2 – 0.7 V vs Hg/HgO reference electrode in 1 M KOH for 500 cycles. 24

Table S1 – Zeta potential values for the AMO-Ni_3Fe-borate-LDH, Li_xMoS_2 (Nanosheet), Li_xWS_2 (Nanosheet) and the hybrid materials MoS_2/Ni_3Fe-LDH and WS_2/Ni_3Fe-LDH measured at a concentration of 1 mg mL^{-1}. 7

Table S2 – Fitted resistance results from Nyquist plots in Figure S12 for; the MoS_2/Ni_3Fe-LDH and WS_2/Ni_3Fe-LDH hybrid materials, and the corresponding TMD and LDH nanosheet precursors – AMO-Ni_3Fe-borate-LDH, Li_xMoS_2 (Nanosheet), and Li_xWS_2 (Nanosheet). "C-Ni_3Fe-A-LDH"; A = carbonate or borate, denotes the conventionally synthesised LDH and "(PM)" denotes the physical mixture of TMD and LDH. 17

Table S3 – OER performance summary of comparative LDH materials interstratified with different 2D substrate materials recently reported for basic solutions. 25

Electrode Preparation

The working electrodes were prepared by drop-casting a “catalyst ink” onto the surface of a glassy carbon electrode. The catalyst ink was prepared by mixing 1 mL of sample solution (5 mg mL^{-1}) with 25 µL of 5 % Aquivion® (Sigma Aldrich, 25 % in water, then diluted) solution and sonicating for 25 min. The glassy carbon electrode was prepared by polishing with 1 µm alumina slurries to obtain a mirror finish. The electrodes were rinsed thoroughly with MilliQ water (18 MΩ cm), before sonicating in MilliQ water for 10 min, and then dried under ambient conditions. Finally, 5 µL of this ink (24.4 µg of catalyst) was dropped onto the centre of the glassy carbon electrode (0.07 cm^2) and allowed to dry in ambient air to form the catalyst film. Catalyst films were inspected visually before and after electrochemical testing for evidence of cracking, delamination, or non-uniform coverage.

Electrochemical Measurements

Electrochemical OER measurements were acquired using a Gamry Reference 3000 Potentiostat in a 3-electrode set-up; Hg/HgO reference electrode (1.0 M KOH), Pt counter electrode (1 cm^2) and a glassy-carbon (GC) working electrode (d = 3 mm), in 1.0 M aqueous solution of potassium hydroxide (KOH; pellets; Sigma Aldrich, ≥85 %) at 25 °C in a glass cell. Prior to measurements, the electrolyte (100 mL) was purged with N_2 gas for 0.5 h, and then a nitrogen blanket was maintained during measurements. All potentials were measured against a Hg/HgO reference electrode with a 1 M KOH internal electrolyte. Potentials were converted relative to the reversible hydrogen electrode (RHE) using:[1-3]

$$E_{RHE} = E_{Hg/HgO} + 0.059 \cdot pH + E^{\Theta}_{Hg/HgO}$$

For 1 M KOH (pH ~ 14), this corresponds to:

$$E_{RHE} = E_{Hg/HgO} + 0.925 \text{ V}$$

The uncompensated solution resistance (R_u) was determined before each measurement and the potentials were corrected for this ohmic loss using:[4]

$$E_{iR} = E_{RHE} - iR_u$$

Electrochemical data were collected from five independently prepared electrodes, with at least three LSV measurements performed per prepared electrode. A cyclic voltammetry (CV) scan was performed from 0.925 – 1.625 V *vs* V_{RHE} at 100 mV s^{-1} to activate the prepared catalysts, for at least 15 cycles until a stable current density was achieved. Linear sweep voltammetry (LSV) scans were obtained at a scan rate of 5 mV s^{-1} over the potential range 1.125 – 1.725 V *vs* V_{RHE}. Reported overpotentials and Tafel slopes represent the mean values ± standard error, accounting for both preparation and measurement variability. The overpotential required to achieve a current density of 10 mA cm^{-2}, η_{10}, was calculated according to:[5]

$$\eta_{10} = E_{RHE}(j = 10 \text{ mA cm}^{-2}) - 1.23 \text{ V}$$

Electrochemical impedance spectroscopy (EIS) was collected at 1.525 V *vs* V_{RHE} over a frequency range of 100000 – 0.1 Hz, with an alternating current (AC) perturbation amplitude of 10 mV. Using an equivalent circuit model, the solution resistance (R_s), interfacial resistance (R_f), and charge transfer resistance (R_{ct}) were extracted and are detailed in Table S2.[6, 7]

Electrochemical surface area (ECSA) was determined from the electrochemical double-layer capacitance (C_{dl}). CV scans were measured in the non-Faradaic potential window 0.975 – 1.125 V *vs* V_{RHE}. The absence of distinct redox features in this region was verified separately for each catalyst. CV scans were collected at scan rates of 5, 10, 20, 50, 100, 200, 400 mV s^{-1}, although only the scan-rate range over which a linear capacitive response was observed was used for the final regression. The capacitive current, j_{capa}, was calculated at the midpoint of the scan at 1.05 V *vs* V_{RHE} as:

$$j_{\text{capa}} = \frac{j_a - j_c}{2}$$

The slope of plot between j_{capa} and scan rate yielded the C_{dl} (μF cm^{-2}). ECSA was estimated by dividing the C_{dl} by the literature value for the specific capacitance, C_s, of a transition metal hydroxide (40 μF cm^{-2}).[3] However, the true specific capacitance depends on composition, surface roughness, electrolyte and applied potential, and thus the resulting ECSA values are treated as approximate comparative estimates rather than absolute surface areas. Measurements were repeated three times per independently prepared electrode, and the mean values ± standard error are reported, accounting for both preparation and measurement variability.

Electrochemical OER stability was evaluated by chronopotentiometry (CP) and accelerated durability testing (ADT) over 500 CV cycles:

- Comparative CP measurements were collected under a constant current density of 10 mA cm^{-2} for 12 h, with stirring at 700 rpm to mitigate bubble formation over the working electrode surface. Separate extended CP measurements of the two hybrid materials, using independently prepared catalyst-coated electrodes, were measured until loss of stable operation. Evaporation was negligible and the reference electrode was checked before and after testing.
- ADT was performed by cycling the potential across 1.125 – 1.625 V at a scan rate of 100 mV s^{-1} for 500 cycles. LSV and Tafel slopes were recorded before and after CP and ADT measurements to assess changes in intrinsic catalytic activity.

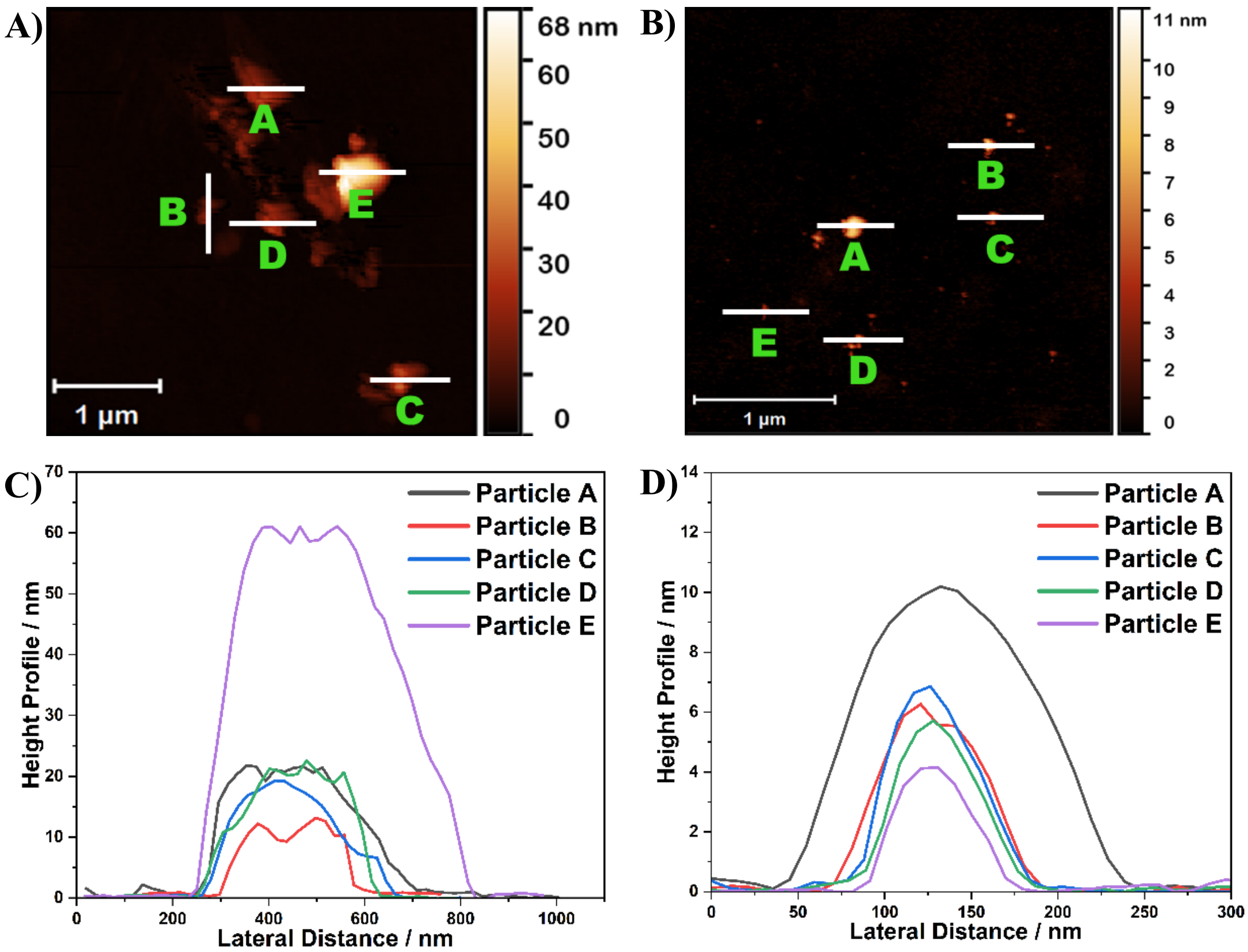

Figure S1 – AFM images of **A)** the MoS_2/Ni_3Fe-LDH hybrid and **B)** the WS_2/Ni_3Fe-LDH hybrid and the corresponding height profile cross-sections associated with each of the labelled particles; **C)** and **D)**.

Table S1 – Zeta potential values for the AMO-Ni_3Fe-borate-LDH, Li_xMoS_2 (Nanosheet), Li_xWS_2 (Nanosheet) and the hybrid materials MoS_2/Ni_3Fe-LDH and WS_2/Ni_3Fe-LDH measured at a concentration of 1 mg mL^{-1}.

Sample	Zetapotential / mV	PDI
**AMO-Ni_3Fe-borate-LDH**	36.50	0.01
**Li_xMoS_2 (Nanosheet)**	–35.80	0.02
**MoS_2/Ni_3Fe-LDH**	–1.80	2.65
**Li_xWS_2 (Nanosheet)**	–33.00	0.03
**WS_2/Ni_3Fe-LDH**	–1.67	3.34

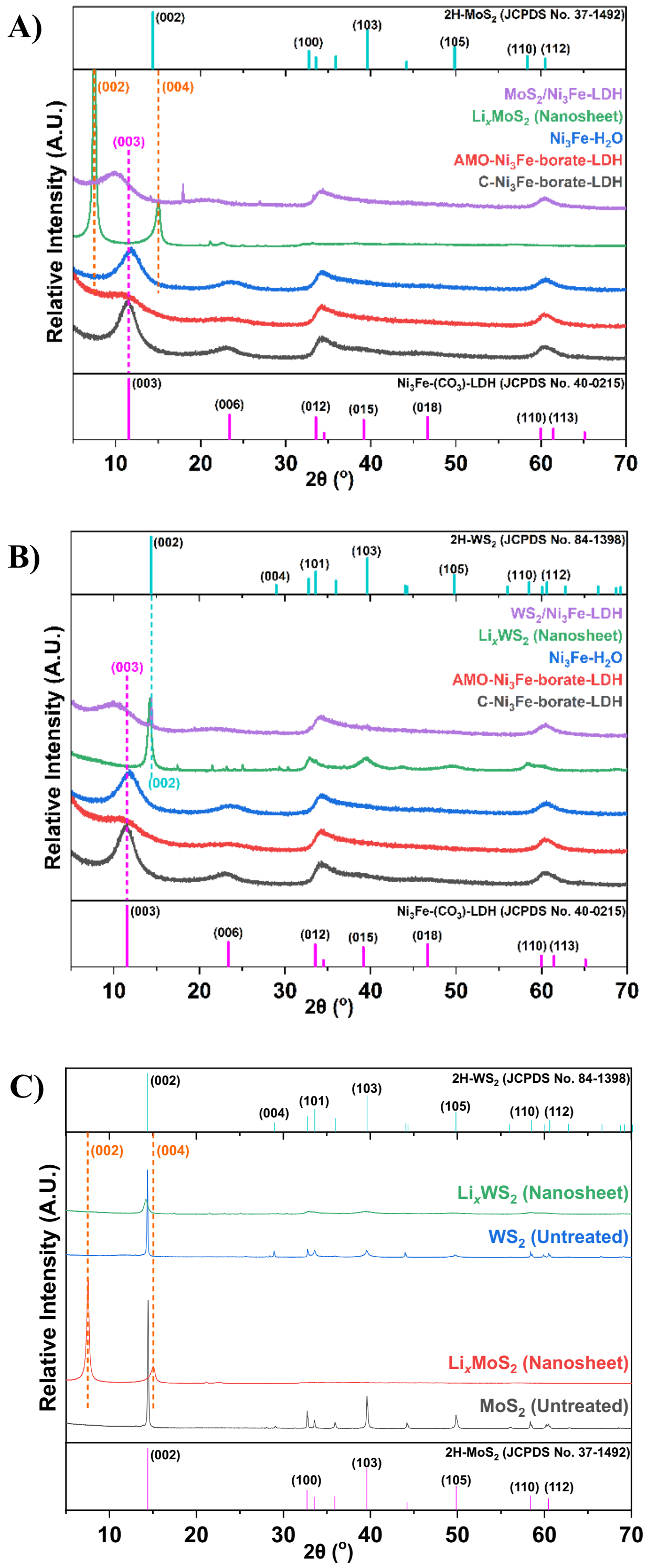

Figure S2 – PXRD patterns for **A)** the MoS_2/Ni_3Fe-LDH hybrid (green line) and corresponding TMD and LDH precursors – Li_xMoS_2 (Nanosheet) (gold line) and AMO-Ni_3Fe-borate-LDH (red line), respectively, and **B)** the corresponding data for the WS_2/Ni_3Fe-LDH hybrid. "(PM)" denotes the physical mixture of TMD and LDH, and Ni_3Fe-H_2O denotes the simple water re-stacked material (blue line). **C)** PXRD patterns for the untreated, bulk TMD powders and the corresponding Li_xMS_2 (Nanosheet) materials.

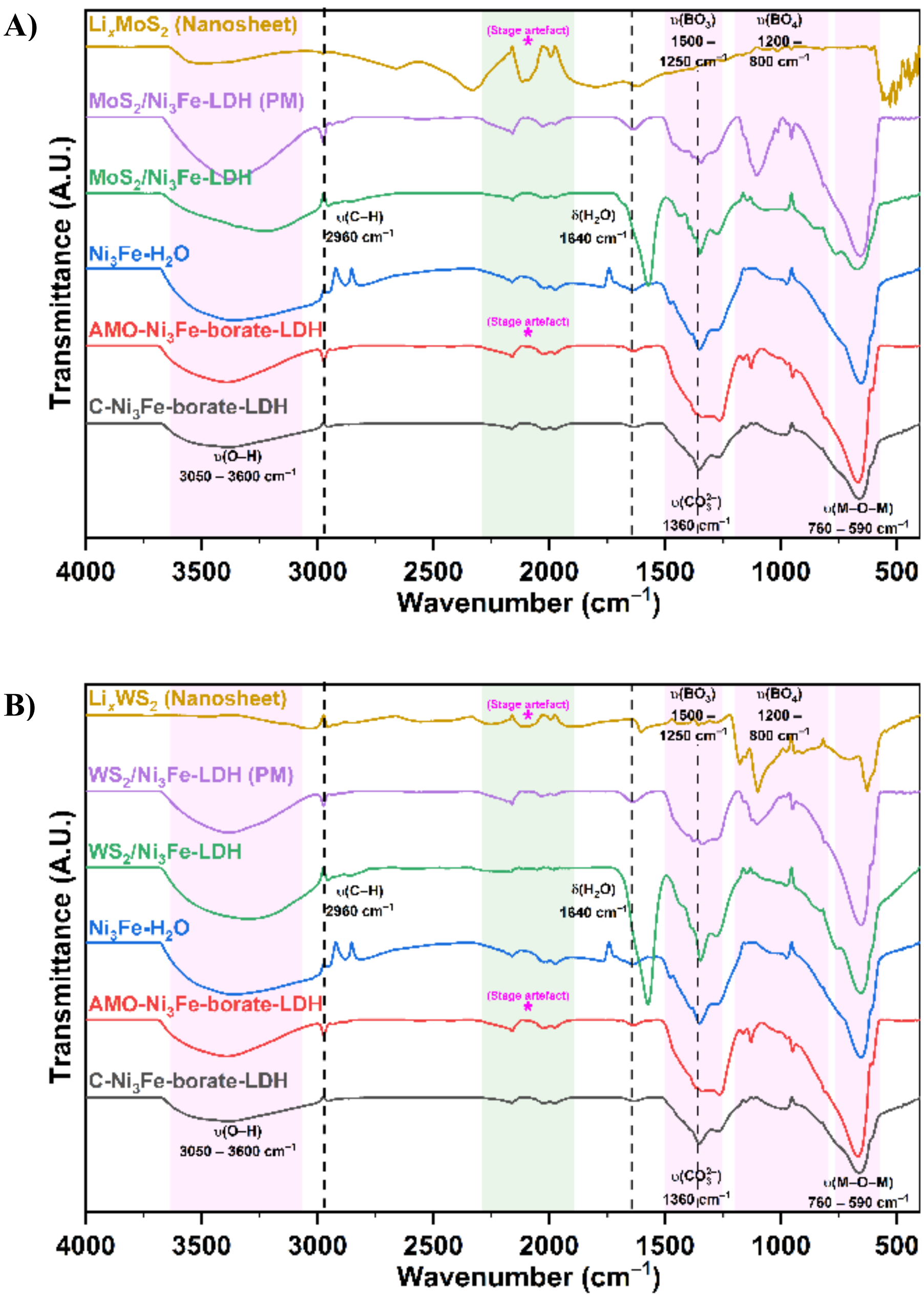

Figure S3 – FTIR spectra for **A)** the MoS_2/Ni_3Fe-LDH hybrid (green line) and corresponding TMD and LDH precursors – Li_xMoS_2 (Nanosheet) (gold line) and AMO-Ni_3Fe-borate-LDH (red line), respectively, and **B)** the corresponding data for the WS_2/Ni_3Fe-LDH hybrid. "(PM)" denotes the physical mixture of TMD and LDH, and Ni_3Fe-H_2O denotes the simple water re-stacked material (blue line). The region denoted by * is an artefact of the sample stage.

A)

B)

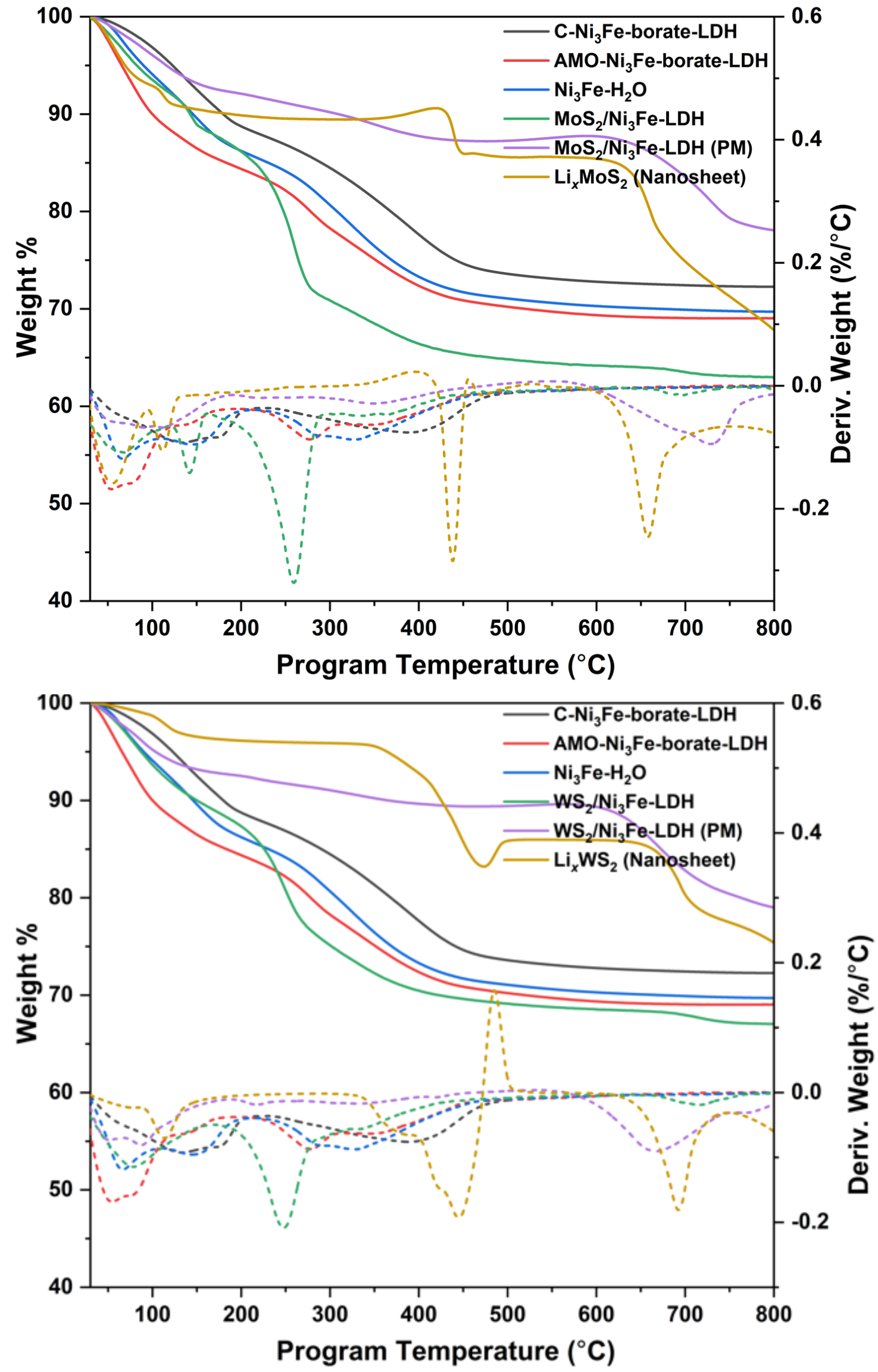

Figure S4 – TGA curves for **A)** the MoS_2/Ni_3Fe-LDH hybrid (green line) and corresponding TMD and LDH precursors – Li_xMoS_2 (Nanosheet) (gold line) and AMO-Ni_3Fe-borate-LDH (red line), respectively, and **B)** the corresponding data for the WS_2/Ni_3Fe-LDH hybrid. "(PM)" denotes the physical mixture of TMD and LDH, and Ni_3Fe-H_2O denotes the simple water re-stacked material (blue line). TGA curves were collected under N_2 at a ramp rate of 10 °C min^{-1} with the accompanying differential TGA (dTGA) curves shown as dotted lines.

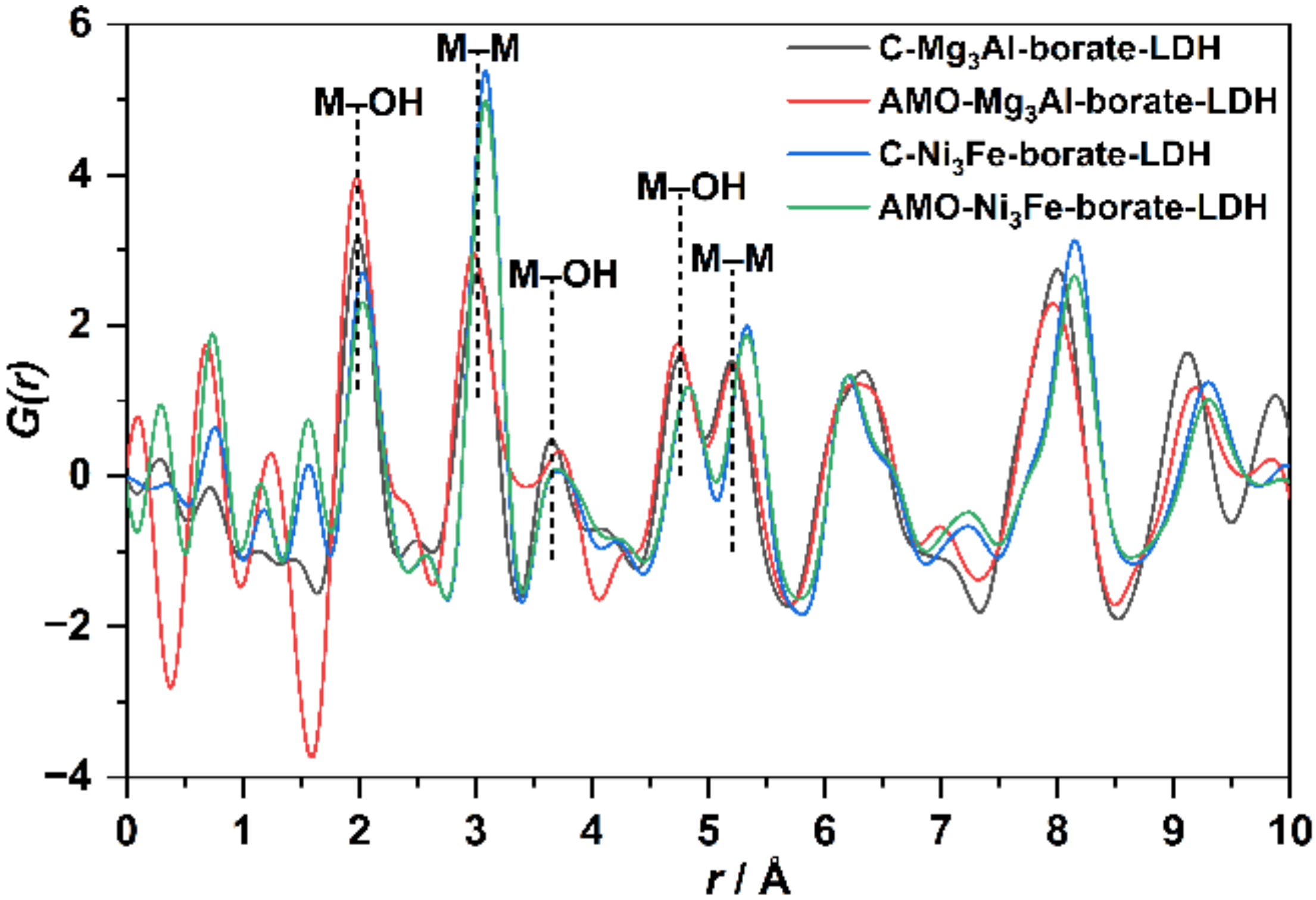

Figure S5 – X-ray pair distribution functions (XPDF) spectra for **A)** Mg_3Al-borate-LDH (black = C-LDH, red = AMO-LDH) and Ni_3Fe-borate-LDHs (blue = C-LDH, green = AMO-LDH) and **B)** AMO-Ni_3Fe-borate-LDH (black line), and the corresponding hybrid materials MoS_2/Ni_3Fe-LDH (red line) and WS_2/Ni_3Fe-LDH (blue line).

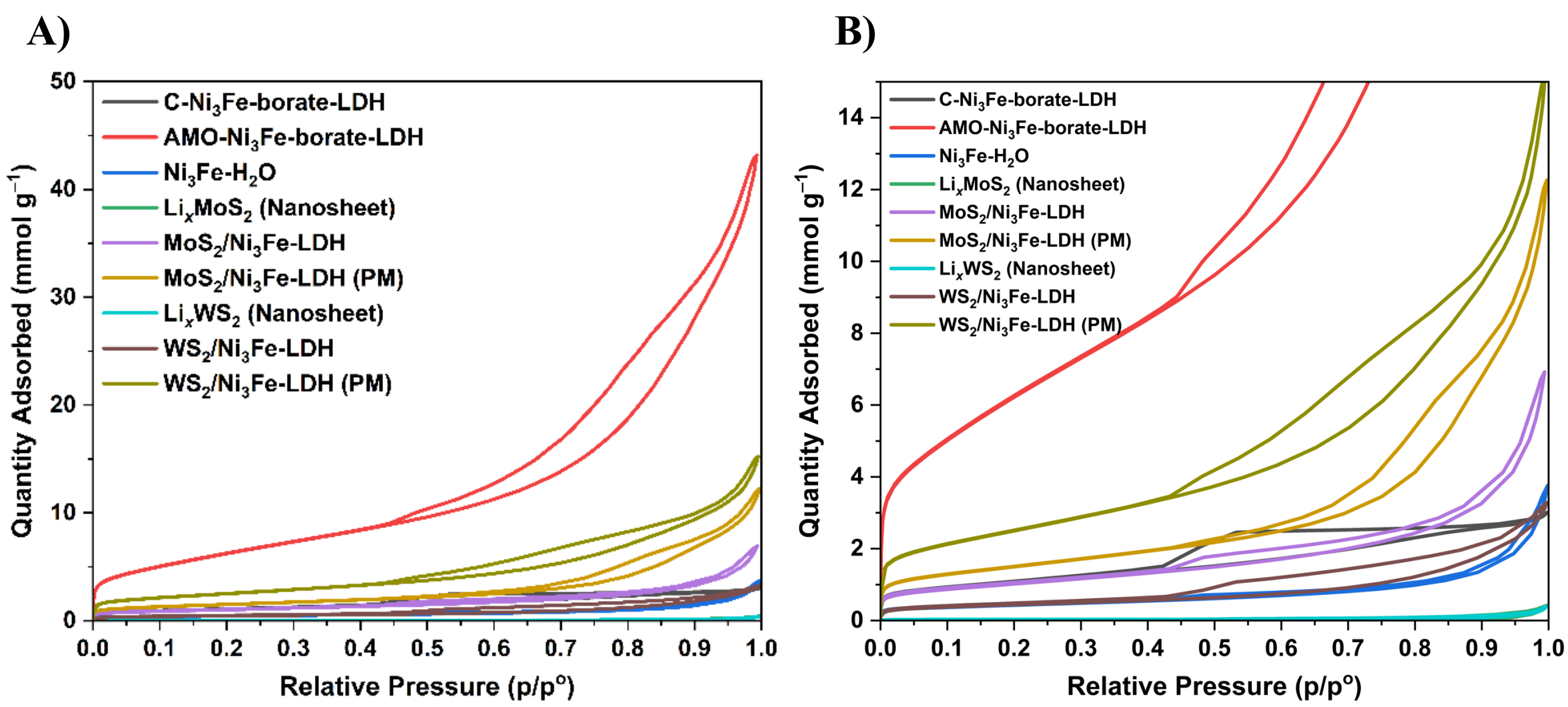

Figure S6 – A) N_2 adsorption-desorption for the MoS_2/Ni_3Fe-LDH hybrid and corresponding TMD and LDH precursors – Li_xMoS_2 (Nanosheet) and AMO-Ni_3Fe-borate-LDH, respectively, and the corresponding data for the WS_2/Ni_3Fe-LDH hybrid. "(PM)" denotes the physical mixture of TMD and LDH, and Ni_3Fe-H_2O denotes the simple water re-stacked material. **B)** Magnified version of **A)** in the *y*-axis.

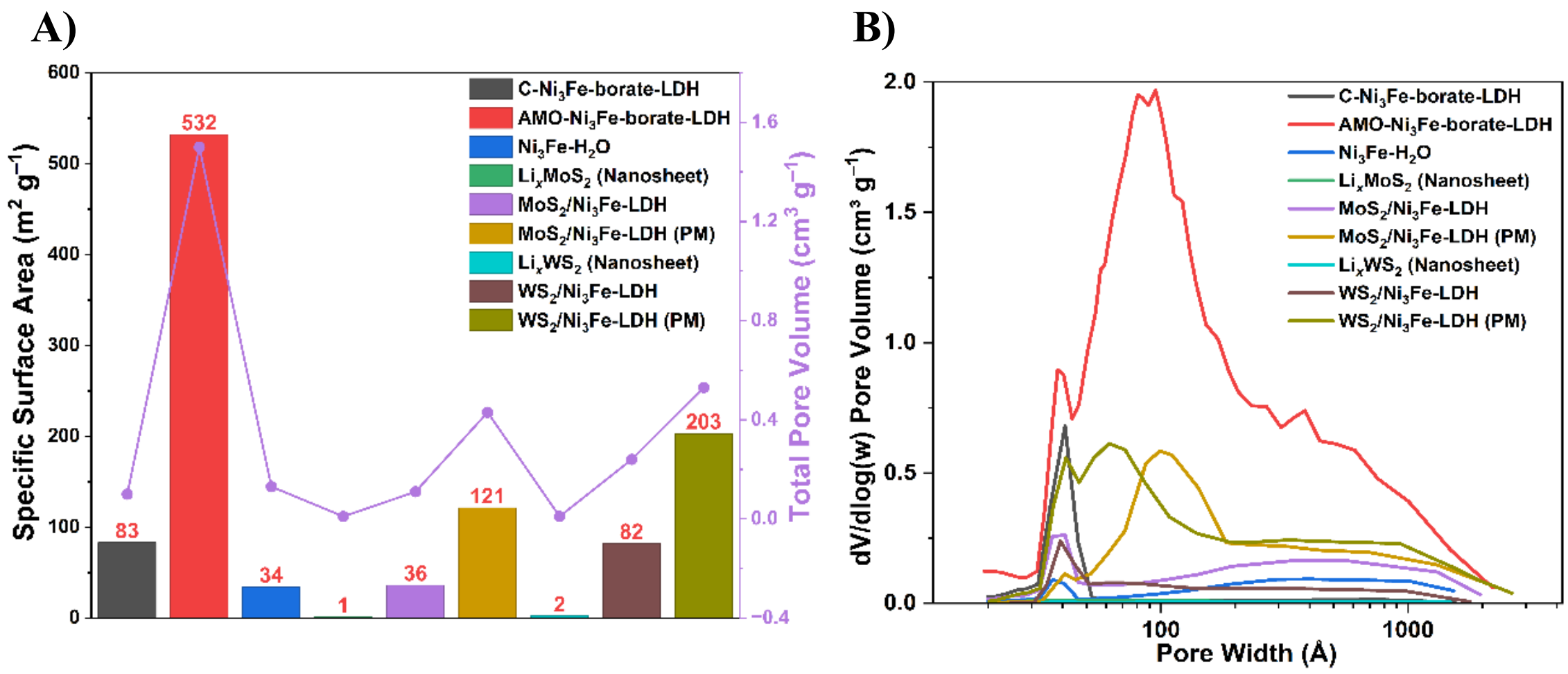

Figure S7 – Summary of surface analysis data for the MoS_2/Ni_3Fe-LDH hybrid and corresponding TMD and LDH precursors – Li_xMoS_2 (Nanosheet) and AMO-Ni_3Fe-borate-LDH, respectively, and the corresponding data for the WS_2/Ni_3Fe-LDH hybrid. "(PM)" denotes the physical mixture of TMD and LDH, and Ni_3Fe-H_2O denotes the simple water re-stacked material. **A)** BET-derived specific surface areas and total pore volumes, and **B)** BJH desorption-derived pore size distributions.

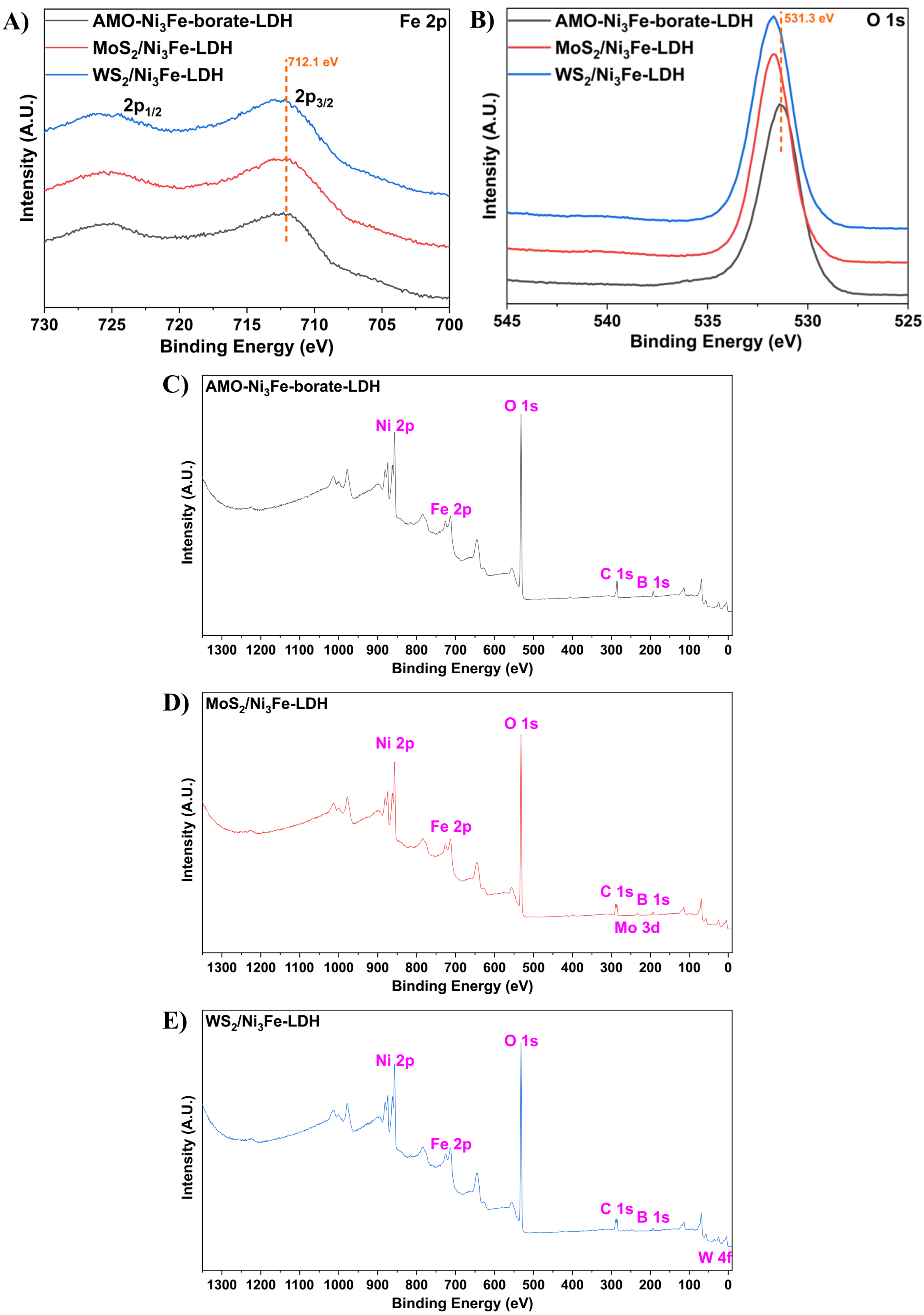

Figure S8 – X-ray photoelectron spectroscopy (XPS) spectra of **A)** Fe 2p and **B)** O 1s for AMO-Ni_3Fe-borate-LDH (black line), and the corresponding hybrid materials MoS_2/Ni_3Fe-LDH (red line) and WS_2/Ni_3Fe-LDH (blue line). XPS survey spectra for **C)** AMO-Ni_3Fe-borate-LDH, **D)** MoS_2/Ni_3Fe-LDH, and **E)** WS_2/Ni_3Fe-LDH.

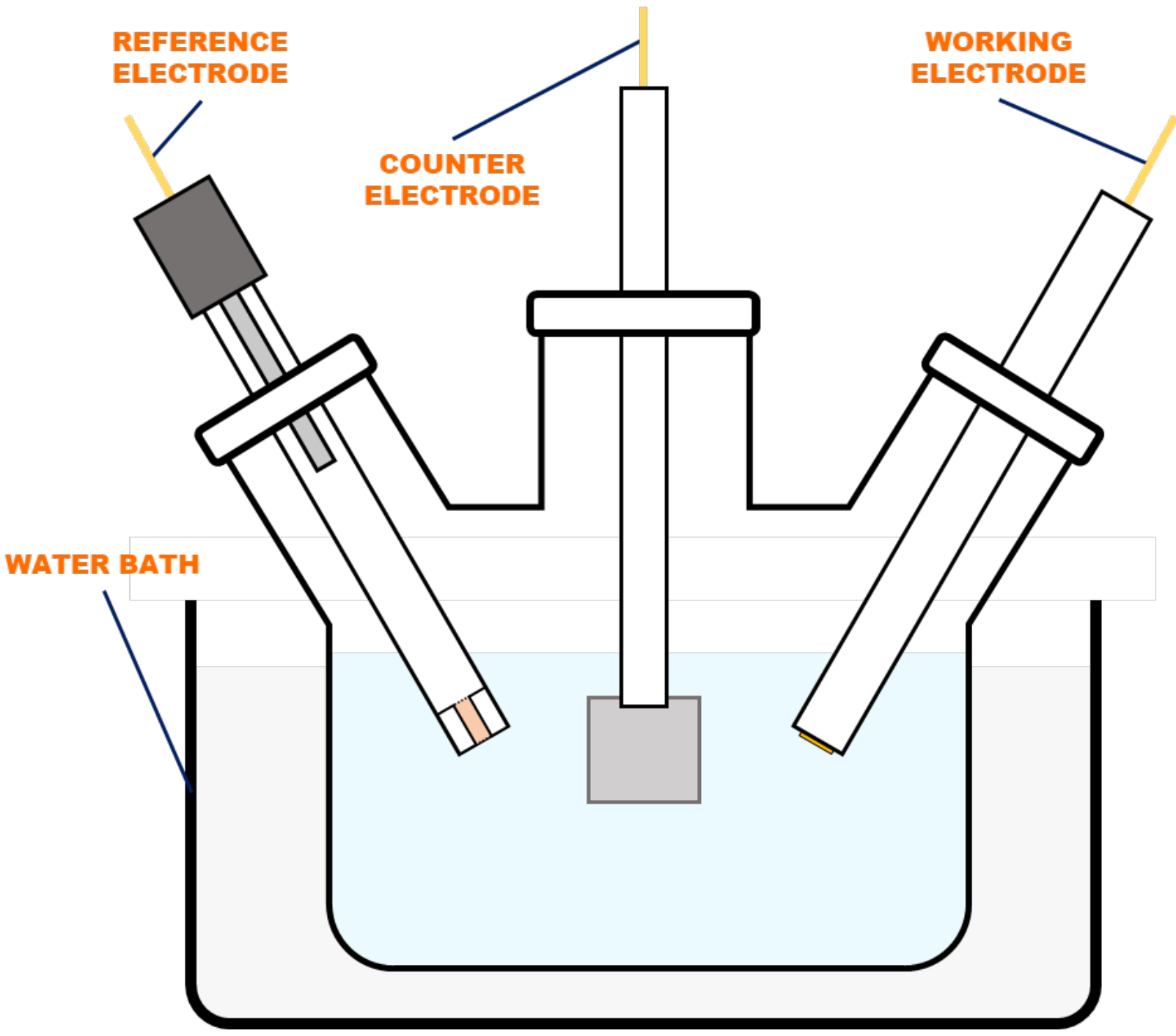

Figure S9 – Experimental 3-electrode setup consisting of Hg/HgO reference electrode (RE), 1 cm^2 Pt counter electrode (CE), and a glassy-carbon working electrode (WE) immersed in 1 M KOH at 25 °C.

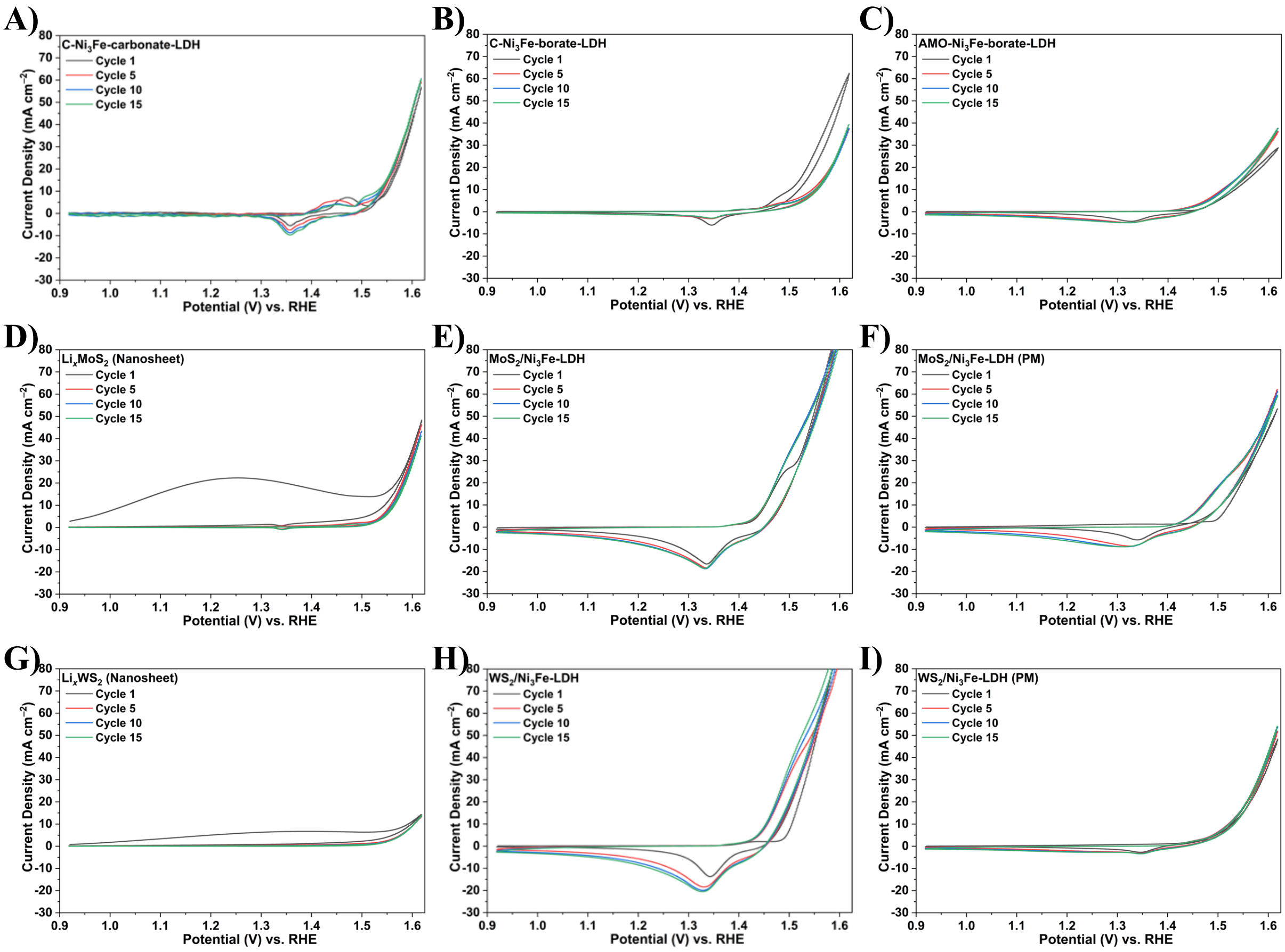

Figure S10 – Cyclic voltammetry (CV) data for; **A)**, **B)** "C-Ni_3Fe-A-LDH"; A = carbonate or borate, denotes the conventionally synthesised LDH, **C)** AMO-Ni_3Fe-borate-LDH, **D)**, **G)** Li_xMoS_2 (Nanosheet) and Li_xWS_2 (Nanosheet), **E)**, **H)** the MoS_2/Ni_3Fe-LDH and WS_2/Ni_3Fe-LDH hybrid materials, and **F)**, **I)** the corresponding physical mixtures of TMD and LDH denoted "(PM)". CV data were recorded at a scan rate of 100 mV s^{-1} in the range: 0.0 – 0.7 V *vs* Hg/HgO reference electrode in 1 M KOH.

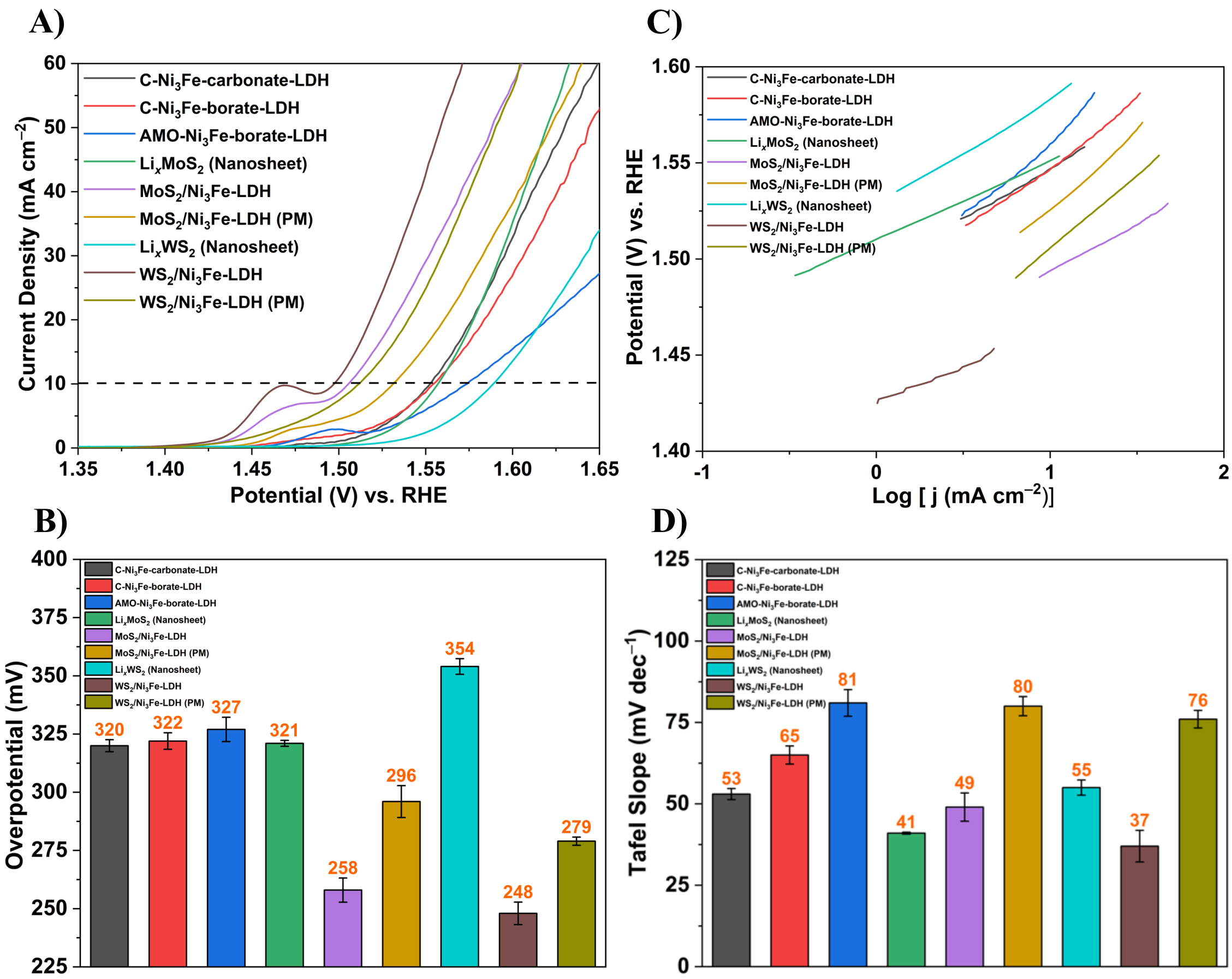

Figure S11 – A) Linear sweep voltammetry (LSV) curves, and **B)** extracted overpotential at a current density of 10 mA cm^{-2}, **C)** Tafel plots for OER, and **D)** Tafel slope extracted from the gradient of the Tafel plots for; the MoS_2/Ni_3Fe-LDH and WS_2/Ni_3Fe-LDH hybrid materials, and the corresponding TMD and LDH nanosheet precursors – AMO-Ni_3Fe-borate-LDH, Li_xMoS_2 (Nanosheet), and Li_xWS_2 (Nanosheet). "C-Ni_3Fe-A-LDH"; A = carbonate or borate, denotes the conventionally synthesised LDH and "(PM)" denotes the physical mixture of TMD and LDH.

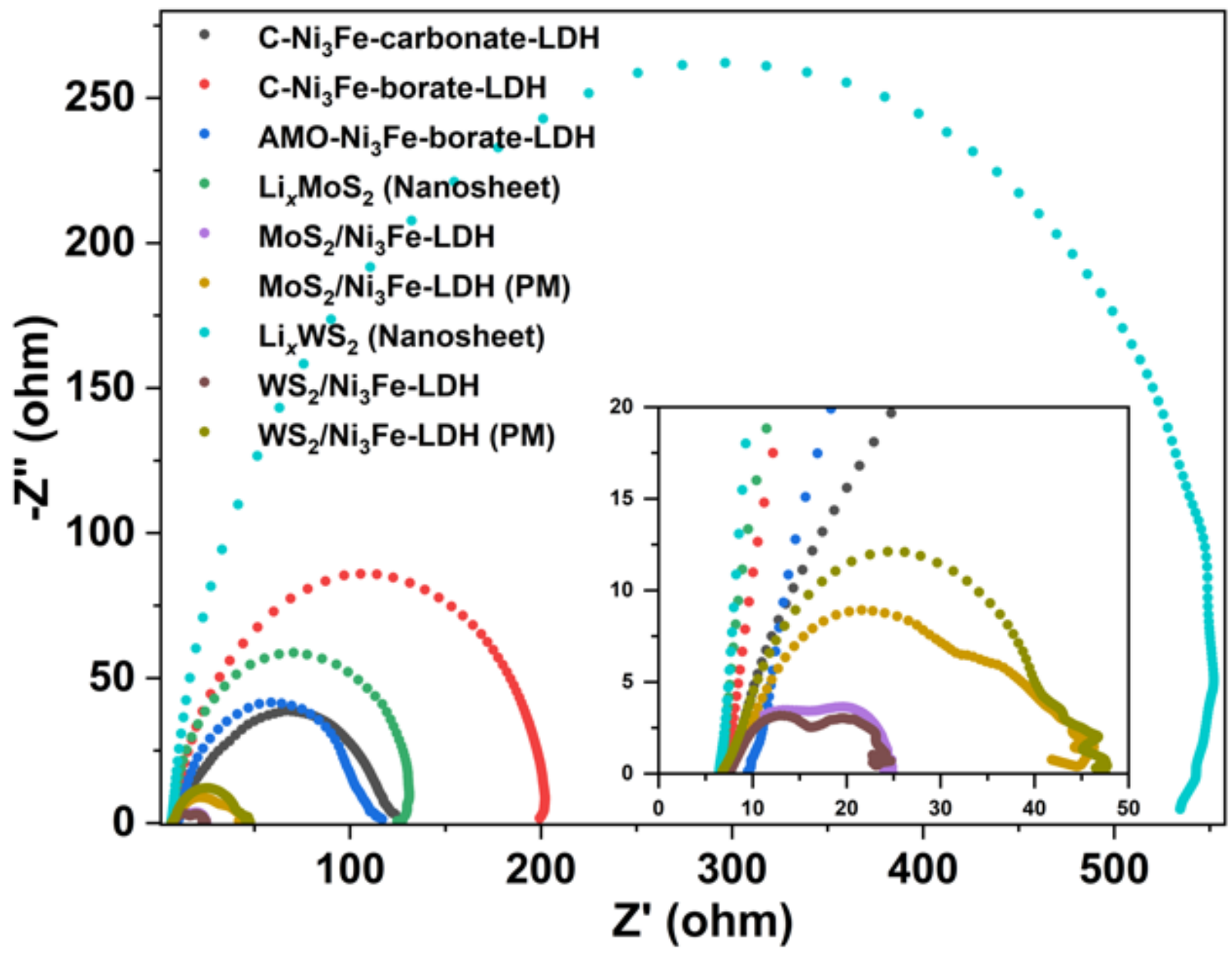

Figure S12 – Electrochemical impedance spectroscopy (EIS) Nyquist plots for; the MoS_2/Ni_3Fe-LDH and WS_2/Ni_3Fe-LDH hybrid materials, and the corresponding TMD and LDH nanosheet precursors – AMO-Ni_3Fe-borate-LDH, Li_xMoS_2 (Nanosheet), and Li_xWS_2 (Nanosheet). "C-Ni_3Fe-A-LDH"; A = carbonate or borate, denotes the conventionally synthesised LDH and "(PM)" denotes the physical mixture of TMD and LDH.

Table S2 – Fitted resistance results from Nyquist plots in Figure S12 for; the MoS_2/Ni_3Fe-LDH and WS_2/Ni_3Fe-LDH hybrid materials, and the corresponding TMD and LDH nanosheet precursors – AMO-Ni_3Fe-borate-LDH, Li_xMoS_2 (Nanosheet), and Li_xWS_2 (Nanosheet). "C-Ni_3Fe-A-LDH"; A = carbonate or borate, denotes the conventionally synthesised LDH and "(PM)" denotes the physical mixture of TMD and LDH.

Sample	R_s / Ω	R_f / Ω	R_{ct} / Ω
**C-Ni_3Fe-carbonate-LDH**	7.7	20.6	91.3
**C-Ni_3Fe-borate-LDH**	6.9	16.5	199.9
**AMO-Ni_3Fe-borate-LDH**	10.5	26.6	52.0
**Li_xMoS_2 (Nanosheet)**	7.2	1.9	143.3
**MoS_2/Ni_3Fe-LDH**	7.0	6.5	11.1
**MoS_2/Ni_3Fe-LDH (PM)**	7.0	7.5	32.4
**Li_xWS_2 (Nanosheet)**	6.6	0.8	421.7
**WS_2/Ni_3Fe-LDH**	7.3	5.8	10.6
**WS_2/Ni_3Fe-LDH (PM)**	6.9	18.6	24.5

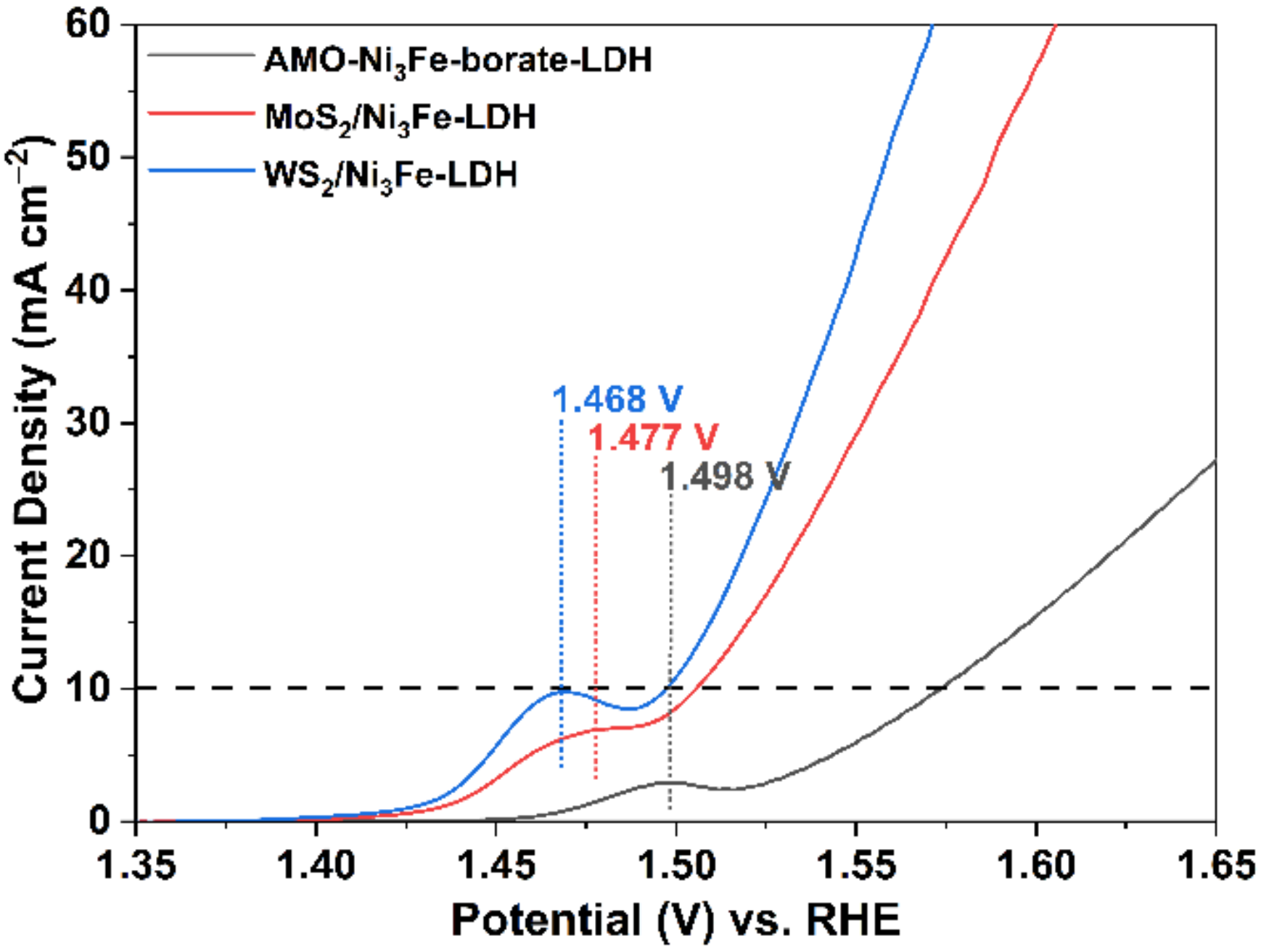

Figure S13 – LSV data for the MoS_2/Ni_3Fe-LDH and WS_2/Ni_3Fe-LDH hybrid materials, and the corresponding AMO-Ni_3Fe-borate-LDH measured in 1 M KOH, highlighting the potential of the Ni redox peak prior to the OER onset potential.

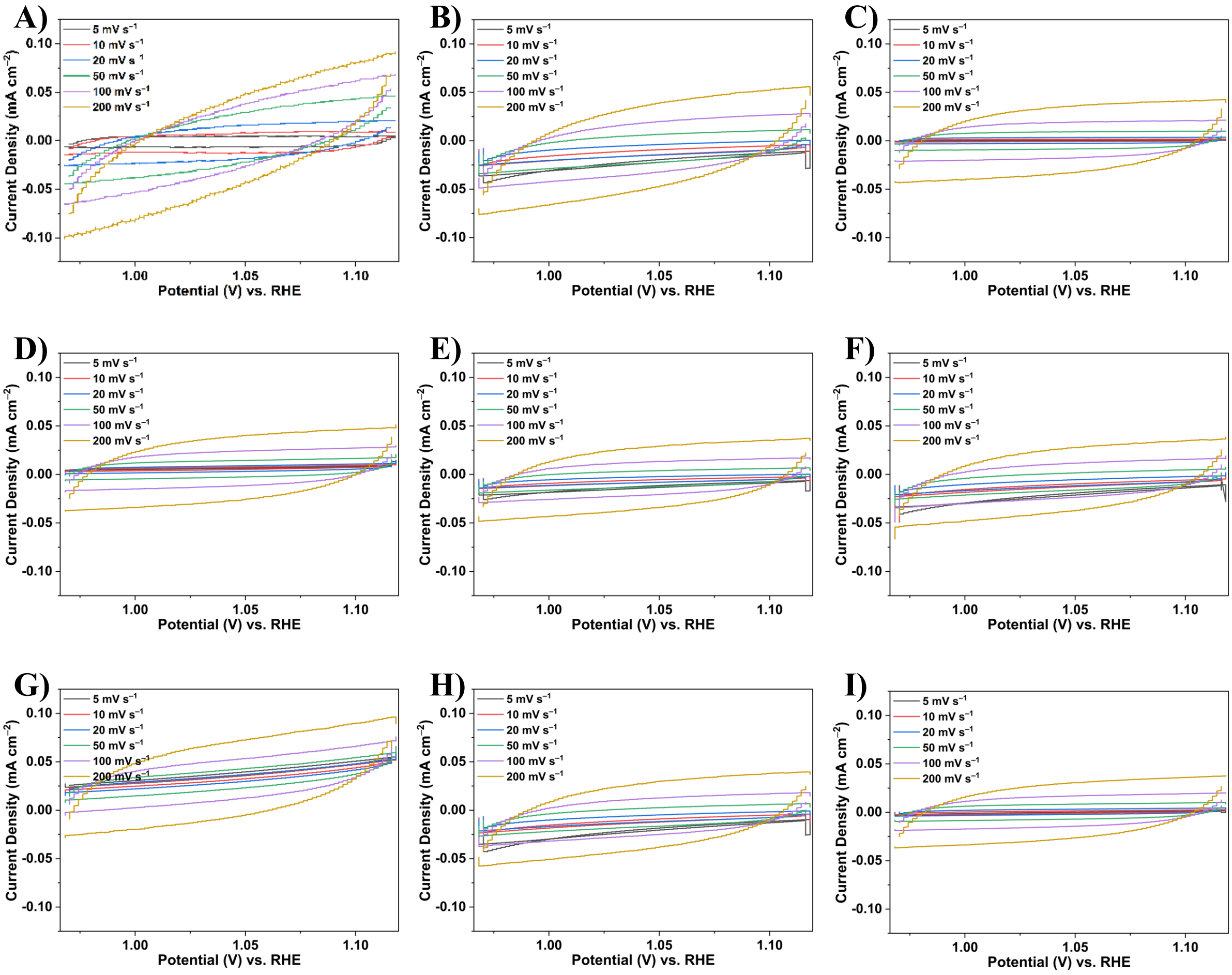

Figure S14 – Cyclic voltammetry (CV) scans at scan rates of 5, 10, 20, 50, 100, and 200 mV s^{-1} for; **A)**, **B)** "C-Ni_3Fe-A-LDH"; A = carbonate or borate, denotes the conventionally synthesised LDH, **C)** AMO-Ni_3Fe-borate-LDH, **D)**, **G)** Li_xMoS_2 (Nanosheet) and Li_xWS_2 (Nanosheet), **E)**, **H)** the MoS_2/Ni_3Fe-LDH and WS_2/Ni_3Fe-LDH hybrid materials, and **F)**, **I)** the corresponding physical mixtures of TMD and LDH denoted "(PM)".

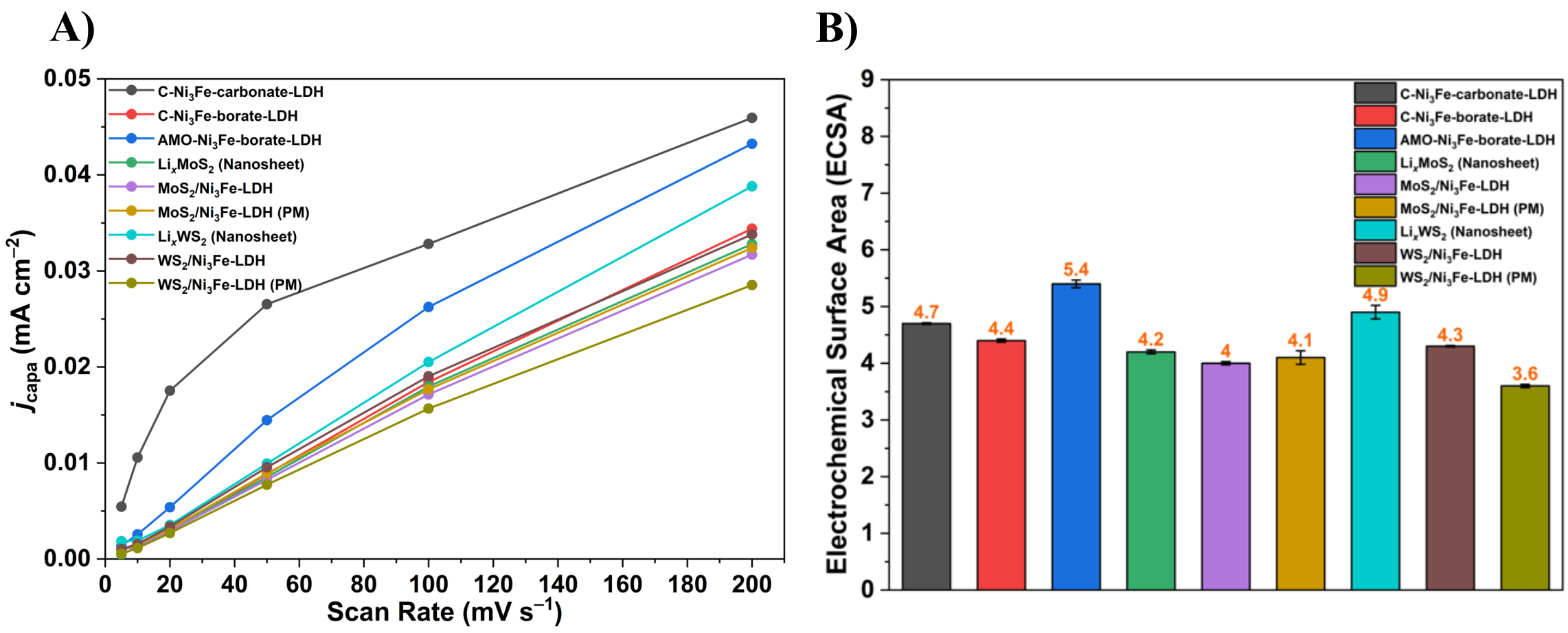

Figure S15 – A) Plot of capacitive current, j_{capa}, against scan rate, and **B)** electrochemical surface area (ECSA) measured from cyclic voltammetry (CV) scans at scan rates of 5, 10, 20, 50, 100, and 200 mV s^{-1} for; the MoS_2/Ni_3Fe-LDH and WS_2/Ni_3Fe-LDH hybrid materials, and the corresponding TMD and LDH nanosheet precursors – AMO-Ni_3Fe-borate-LDH, Li_xMoS_2 (Nanosheet), and Li_xWS_2 (Nanosheet). "C-Ni_3Fe-A-LDH"; A = carbonate or borate, denotes the conventionally synthesised LDH and "(PM)" denotes the physical mixture of TMD and LDH.

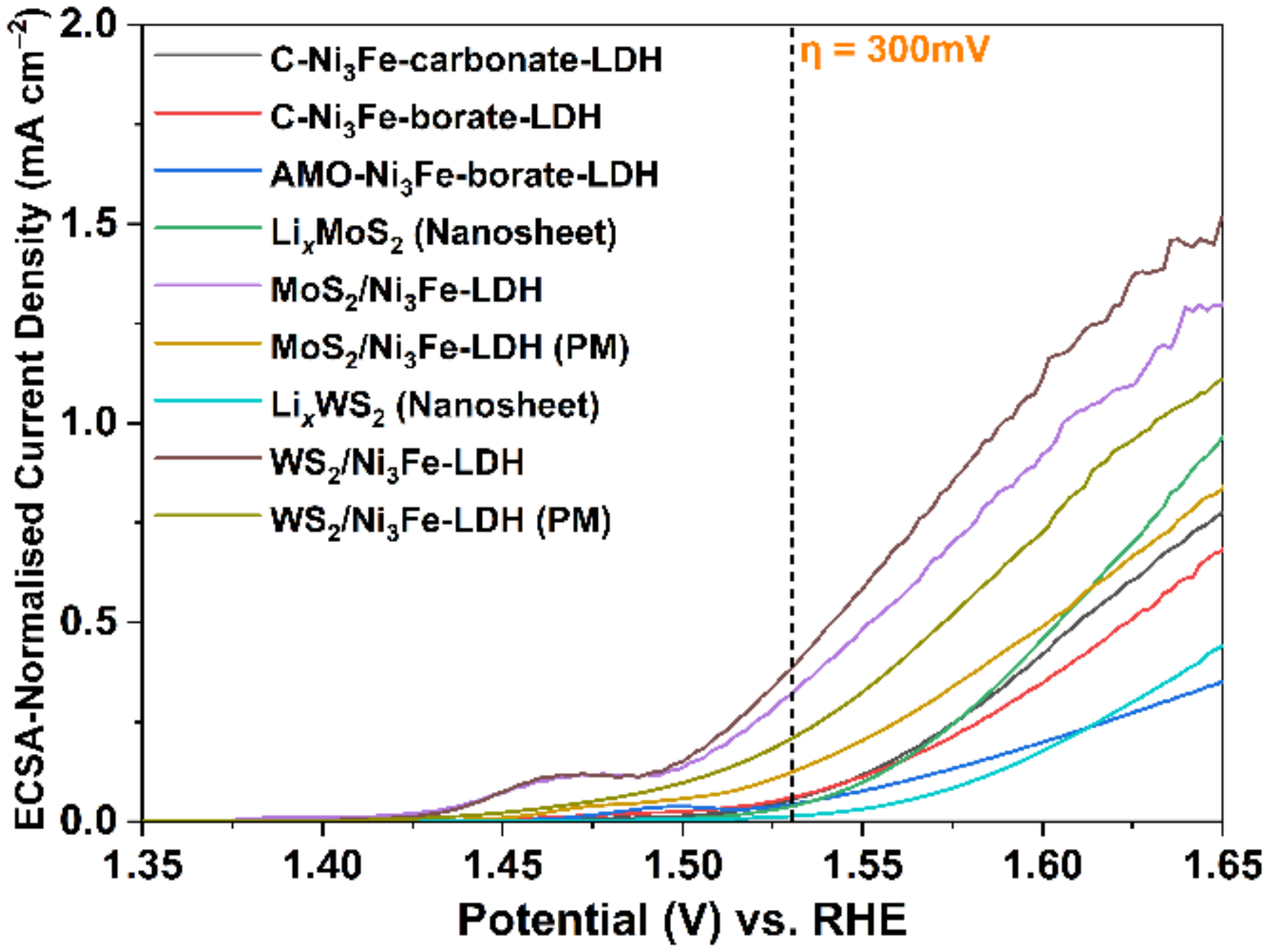

Figure S16 – LSV data normalised by dividing by the ECSA for; the MoS_2/Ni_3Fe-LDH and WS_2/Ni_3Fe-LDH hybrid materials, and the corresponding TMD and LDH nanosheet precursors – AMO-Ni_3Fe-borate-LDH, Li_xMoS_2 (Nanosheet), and Li_xWS_2 (Nanosheet). "C-Ni_3Fe-A-LDH"; A = carbonate or borate, denotes the conventionally synthesised LDH and "(PM)" denotes the physical mixture of TMD and LDH.

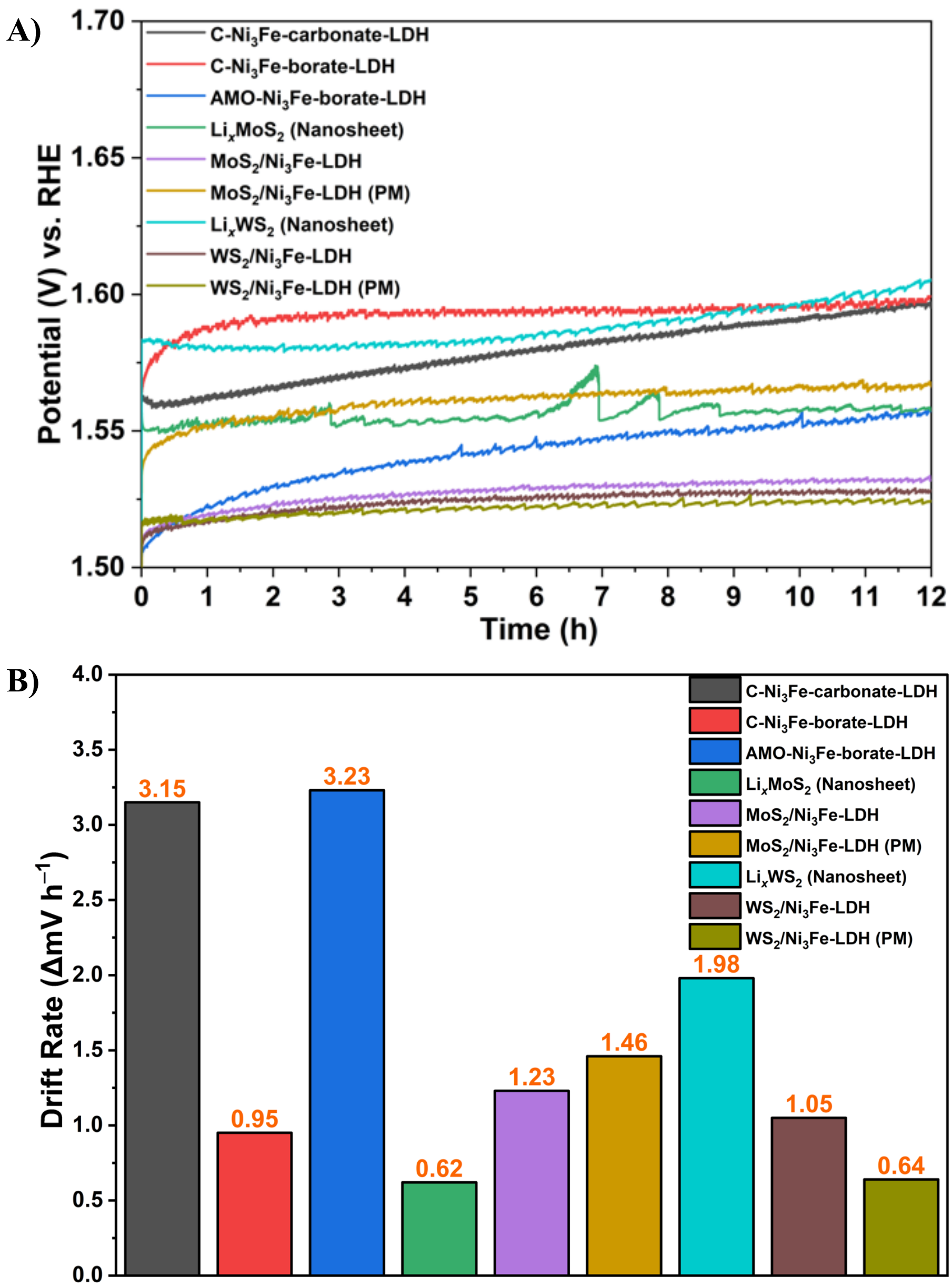

Figure S17 – A) Chronopotentiometry (CP) data at a current density of 10 mA cm^{-2} for 12 h, and **B)** the average potential drift rate measured from the gradient between $t = 0$ and $t = 12$; the MoS_2/Ni_3Fe-LDH and WS_2/Ni_3Fe-LDH hybrid materials, and the corresponding TMD and LDH nanosheet precursors – AMO-Ni_3Fe-borate-LDH, Li_xMoS_2 (Nanosheet), and Li_xWS_2 (Nanosheet). "C-Ni_3Fe-A-LDH"; A = carbonate or borate, denotes the conventionally synthesised LDH and "(PM)" denotes the physical mixture of TMD and LDH.

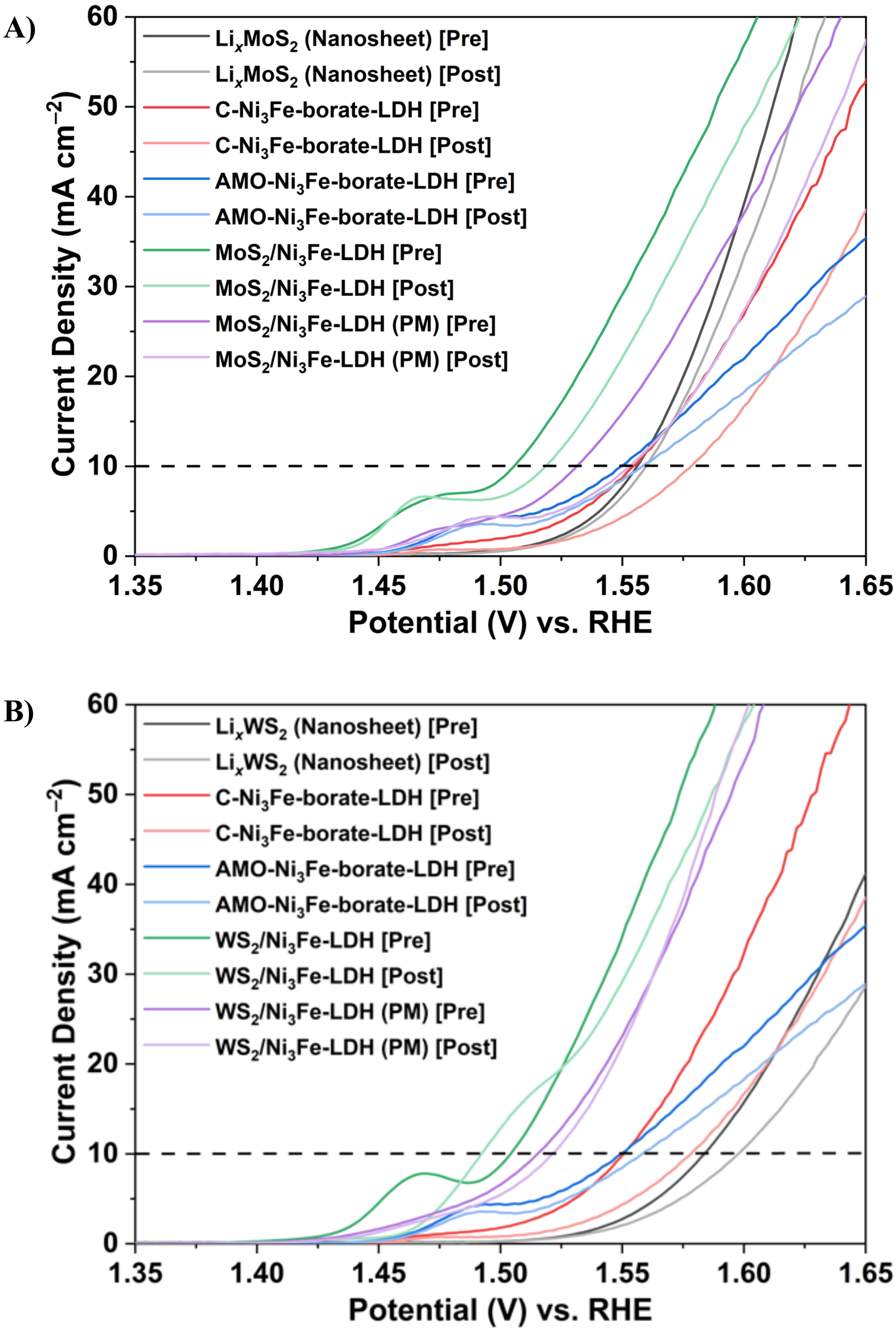

Figure S18 – LSV measured before "[Pre]" and after "[Post]" 12 h CP experiments at 10 mA cm^{-2} for **A)** the MoS_2/Ni_3Fe-LDH hybrid and corresponding TMD and LDH precursors – Li_xMoS_2 (Nanosheet) and AMO-Ni_3Fe-borate-LDH, respectively, and **B)** the corresponding data for the WS_2/Ni_3Fe-LDH hybrid. "(PM)" denotes the physical mixture of TMD and LDH, and Ni_3Fe-H_2O denotes the simple water re-stacked material.

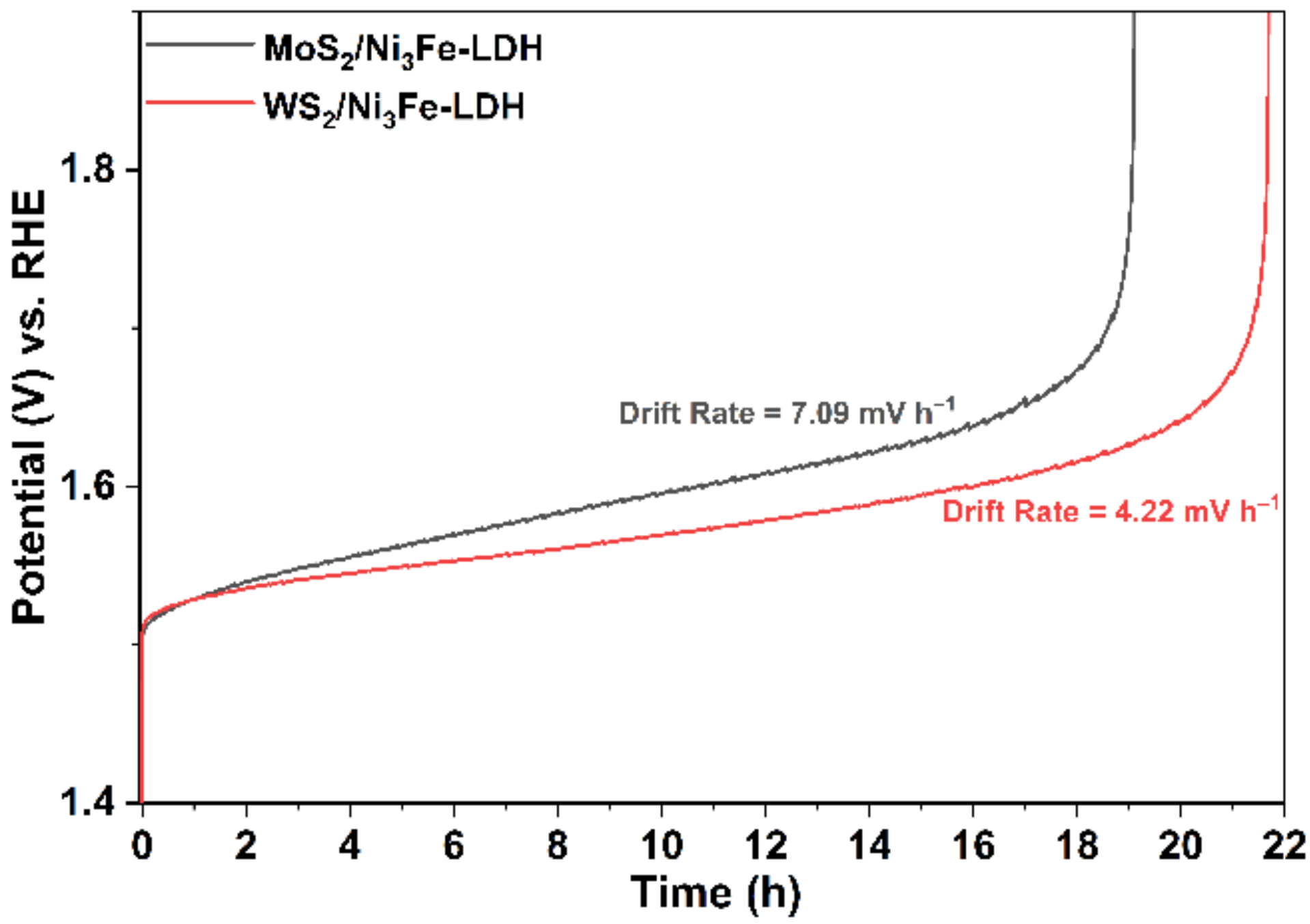

Figure S19 – Chronopotentiometry (CP) data at a current density of 10 mA cm^{-2} for the MoS_2/Ni_3Fe-LDH and WS_2/Ni_3Fe-LDH hybrid materials, measured in independent extended experiments until loss of stable operation. These measurements were performed using separately prepared electrodes and are independent of the 12 h CP measurements shown in Fig. 10 and Fig. S17.

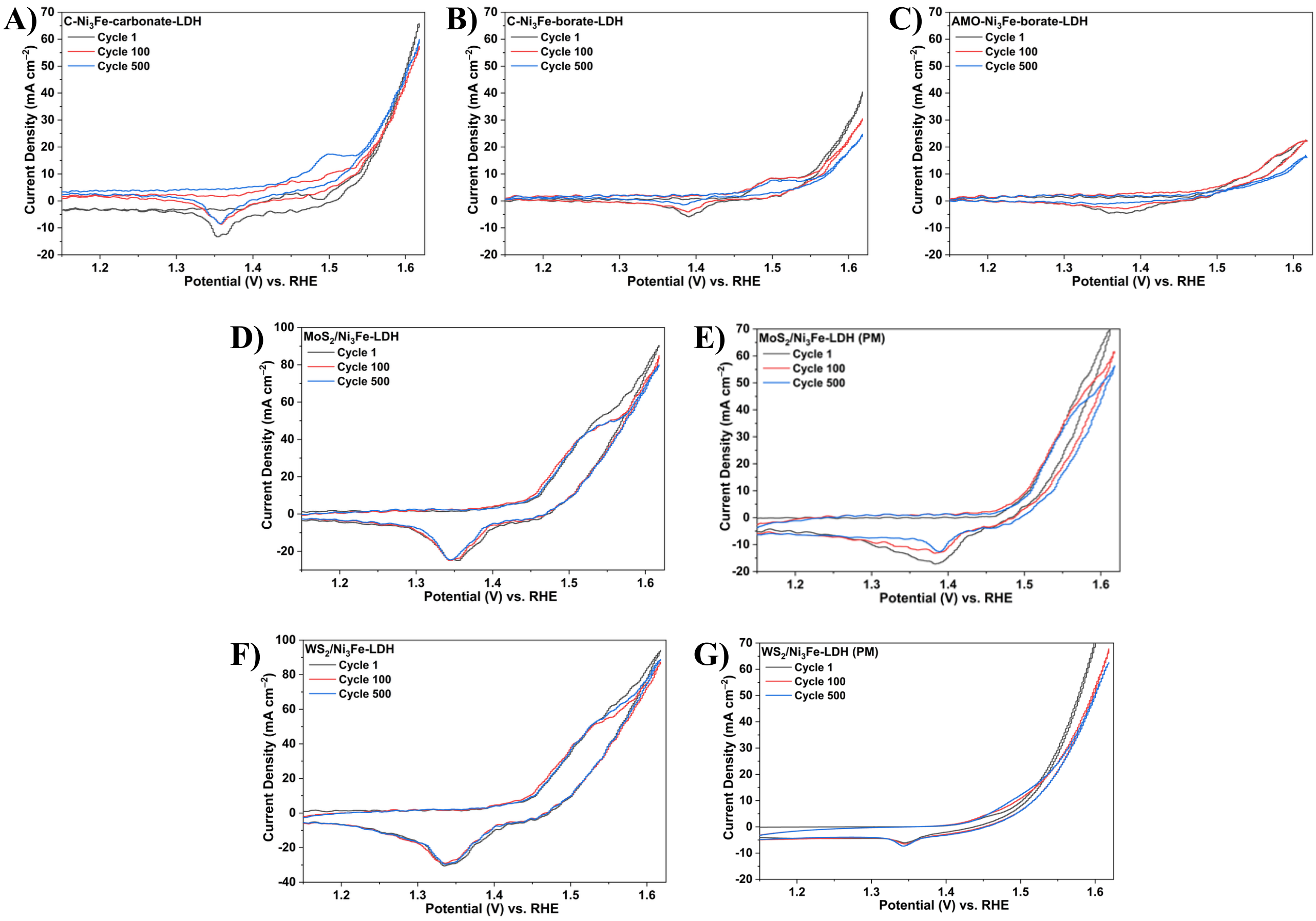

Figure S20 – Accelerated durability testing (ADT) data measured using cyclic voltammetry (CV) for; **A)**, **B)** "C-Ni_3Fe-A-LDH"; A = carbonate or borate, denotes the conventionally synthesised LDH, **C)** AMO-Ni_3Fe-borate-LDH, **D)**, **F)** the MoS_2/Ni_3Fe-LDH and WS_2/Ni_3Fe-LDH hybrid materials, and **E)**, **G)** the corresponding physical mixtures of TMD and LDH denoted "(PM)". CV data were recorded at a scan rate of 100 mV s^{-1} in the range: 0.2 – 0.7 V *vs* Hg/HgO reference electrode in 1 M KOH for 500 cycles.

Table S3 – OER performance summary of comparative LDH materials interstratified with different 2D substrate materials recently reported for basic solutions.

LDH Material	Inter-stratified 2D Material	Hybrid Material Synthesis Method	Overpotential at 10 mA cm^{-2}, η_{10} (mV)	Tafel Slope (mV dec^{-1})	Charge-Transfer Resistance, R_{ct} (Ω)	Chrono-potentiometry Stability at 10 mA cm^{-2} (h)	Ref.
**Ni_2Fe-LDH**	Graphene	**LDH:** Nanosheets formed *via* shaking in formamide for 2 days **Graphene:** Reduced graphene oxide *via* ultrasonication **Hybrid material:** Solution-phase addition with stirring (*no solvent stated*)	230	42	N/A	10	[8]
**NiFe-LDH**	Graphene	**LDH:** Nanosheets formed *via* shaking in formamide **Graphene:** N/A **Hybrid material:** Solution-phase addition with stirring (*no solvent stated*)	220[a]	55[a]	60[a]	N/A	[9]
**Ni_2Fe-LDH**	Graphene	**LDH:** Nanosheets formed *via* stirring in formamide for 2 days **Graphene:** N-doped graphene from pristine monolayer graphene **Hybrid material:** Solution-phase addition with stirring (*no solvent stated*)	210	52	N/A	10	[10]
**Ni_2Fe-LDH**	NaMnO (Birnessite)	**Hybrid material:** Ni and Fe salts co-precipitated directly into a dispersion of NaMnO nanosheets, then aged, centrifuged and freeze-dried	249[a]	21	1.8	N/A	[11]
**MnFe-LDH**	MoS_2	**Hybrid material:** Mn and Fe salts co-precipitated directly into a dispersion of MoS_2 (formed via reduction of MoO_4^{2-} with thiourea)	222	33	1200	25	[12]

**NiFe-LDH**	MoS_2	**LDH:** Nanosheets formed *via* stirring in formamide<br>**TMD:** Nanosheets formed *via* lithiation-aqueous sonication<br>**Hybrid material:** Solution-phase addition with stirring (*no solvent stated*)	210	46	35[a]	10	[9]
**Ni$_3$Al-LDH**	MoS_2	**LDH:** Nanosheets formed *via* stirring in formamide<br>**TMD:** Nanosheets formed *via* lithiation-aqueous sonication<br>**Hybrid material:** Solution-phase addition with stirring (*formamide-aquous mix*)	310	56	159	12	[13]
**Ni$_3$Fe-LDH**	MoS_2	**LDH:** Nanosheets formed *via* stirring in formamide<br>**TMD:** Nanosheets formed *via* lithiation-aqueous sonication<br>**Hybrid material:** Solution-phase addition with stirring (*formamide-aquous mix*)	250	45	49	12	[13]
**Ni$_3$Fe-LDH**	MoS_2	**LDH:** Nanosheets formed *via* AMOST in IPA<br>**TMD:** Nanosheets formed *via* lithiation-aqueous sonication<br>**Hybrid material:** Solution-phase addition with stirring (*formamide-aquous mix*)	258	49	11	19	***This work***
**Ni$_3$Fe-LDH**	WS_2	**LDH:** Nanosheets formed *via* AMOST in IPA<br>**TMD:** Nanosheets formed *via* lithiation-aqueous sonication<br>**Hybrid material:** Solution-phase addition with stirring (*formamide-aquous mix*)	248	37	11	22	***This work***

[a]Approximate values as no values explicity stated in the manuscript or SI.

References

1. S. Q. Niu, S. W. Li, Y. C. Du, X. J. Han and P. Xu, *ACS Energy Lett.*, 2020, **5**, 1083–1087.
2. N. Elgrishi, K. J. Rountree, B. D. McCarthy, E. S. Rountree, T. T. Eisenhart and J. L. Dempsey, *J. Chem. Educ.*, 2018, **95**, 197–206.
3. D. M. Morales and M. Risch, *J. Phys. Energy*, 2021, **3**, 034013.
4. C. Wei, R. R. Rao, J. Peng, B. Huang, I. E. L. Stephens, M. Risch, Z. J. Xu and Y. Shao-Horn, *Adv. Mater.*, 2019, **31**, 1806296.
5. C. C. L. McCrory, S. H. Jung, J. C. Peters and T. F. Jaramillo, *J. Am. Chem. Soc.*, 2013, **135**, 16977–16987.
6. W. Ma, R. Z. Ma, J. H. Wu, P. Z. Sun, X. H. Liu, K. C. Zhou and T. Sasaki, *Nanoscale*, 2016, **8**, 10425–10432.
7. X. P. Huang, K. H. Kim, H. Jang, X. N. Luo, J. F. Yu, Z. Q. Li, Z. M. Ao, J. X. Wang, H. Zhang, C. P. Chen and D. O'Hare, *ACS Appl. Mater. Interfaces*, 2023, **15**, 53815–53826.
8. W. Ma, R. Z. Ma, C. X. Wang, J. B. Liang, X. H. Liu, K. C. Zhou and T. Sasaki, *ACS Nano*, 2015, **9**, 1977–1984.
9. P. Xiong, X. Y. Zhang, H. Wan, S. J. Wang, Y. F. Zhao, J. Q. Zhang, D. Zhou, W. C. Gao, R. Z. Ma, T. Sasaki and G. X. Wang, *Nano Lett.*, 2019, **19**, 4518–4526.
10. Y. Jia, L. Zhang, G. Gao, H. Chen, B. Wang, J. Zhou, M. T. Soo, M. Hong, X. Yan, G. Qian, J. Zou, A. Du and X. Yao, *Adv. Mater.*, 2017, **29**, 1700017.
11. N. Li, Z. Hu, M. Li, L. H. Zhang and S. Hu, *Mater. Lett.*, 2020, **281**, 128569.
12. S. Kumar, S. Raju, S. Marappa, S. Manjunatha, D. R. Vyshak, M. Darshan and H. Bhanupriya, *ACS Appl. Energy Mater.*, 2024, **7**, 9872–9881.
13. M. S. Islam, M. Kim, X. Y. Jin, S. M. Oh, N. S. Lee, H. Kim and S. J. Hwang, *ACS Energy Lett.*, 2018, **3**, 952–960.
